# A Global Transcriptome Analysis Reveals Molecular Hallmarks of Neural Stem Cell Death, Survival, and Differentiation in Response to Partial FGF-2 and EGF Deprivation

**DOI:** 10.1371/journal.pone.0053594

**Published:** 2013-01-07

**Authors:** Vanesa Nieto-Estévez, Jaime Pignatelli, Marcos J. Araúzo-Bravo, Anahí Hurtado-Chong, Carlos Vicario-Abejón

**Affiliations:** 1 Instituto Cajal, Consejo Superior de Investigaciones Científicas (CSIC), Madrid, Spain; 2 Centro de Investigación Biomédica en Red sobre Enfermedades Neurodegenerativas (CIBERNED), Madrid, Spain; 3 Laboratory of Computational Biology and Bioinformatics, Department of Cell and Developmental Biology, Max Planck Institute for Molecular Biomedicine, Muenster, Germany; Universitat Pompeu Fabra, Spain

## Abstract

Neurosphere cell culture is a commonly used model to study the properties and potential applications of neural stem cells (NSCs). However, standard protocols to culture NSCs have yet to be established, and the mechanisms underlying NSC survival and maintenance of their undifferentiated state, in response to the growth factors FGF-2 and EGF are not fully understood. Using cultures of embryonic and adult olfactory bulb stem cells (eOBSCs and aOBSCs), we analyzed the consequences of FGF-2 and EGF addition at different intervals on proliferation, cell cycle progression, cell death and differentiation, as well as on global gene expression. As opposed to cultures supplemented daily, addition of FGF-2 and EGF every 4 days significantly reduced the neurosphere volume and the total number of cells in the spheres, mainly due to increased cell death. Moreover, partial FGF-2 and EGF deprivation produced an increase in OBSC differentiation during the proliferative phase. These changes were more evident in aOBSC than eOBSC cultures. Remarkably, these effects were accompanied by a significant upregulation in the expression of endogenous *Fgf-2* and genes involved in cell death and survival (*Cryab*), lipid catabolic processes (*Pla2g7*), cell adhesion (*Dscaml1*), cell differentiation (*Dscaml1*, *Gpr17*, *S100b, Ndrg2*) and signal transduction (*Gpr17*, *Ndrg2)*. These findings support that a daily supply of FGF-2 and EGF is critical to maintain the viability and the undifferentiated state of NSCs in culture, and they reveal novel molecular hallmarks of NSC death, survival and the initiation of differentiation.

## Introduction

Following closure of the neural tube, neuroepithelial (NE) cells in the vertebrate central nervous system (CNS) divide symmetrically to expand the cell population. Although NE cells can directly differentiate to produce the first neurons in the CNS, the majority transform into radial glial (RG) cells that, following rounds of asymmetric division, generate intermediate progenitors that differentiate into neurons or glia. Accordingly, NE and RG cells in particular represent primary progenitors or neural stem cells (NSCs), and they are best distinguished by their self-renewal abilities, as well as their multipotent differentiation [1], [2], [3], [4], [5]. The completion of cell differentiation during the embryonic and neonatal periods coincides with the exhaustion of NSC populations in all regions of the CNS, except for the adult subventricular zone (SVZ), the subgranular layer of the hippocampal dentate gyrus (DG) [6], [7], [8] and possibly, the olfactory bulb and the hypothalamus [9], [10], [11], [12].

While demonstrating the multipotentiality and the self-renewal capacity of embryonic RG cells and adult RG-derived cells *in vivo* has proved to be technically challenging, retroviral injections, sequential labelling with thymidine analogues, and lineage tracing techniques have demonstrated the existence of these cells in the embryonic and adult brain [4], [6], [13], [14]. However, the full potential of NSCs is more evident when they are seeded as single cells and their clonal expansion is studied in adherent or neurosphere cultures, along with their differentiation into neurons, astrocytes and oligodendrocytes both *in vitro* and after transplantation [11], [15], [16], [17], [18], [19], [20].

It is well established that the addition of both human recombinant fibroblast growth factor-2 (FGF-2) and epidermal growth factor (EGF) (hereafter referred to as FGF-2/EGF) is critical to maintain and expand NSC cultures as floating neurospheres [11], [15], [19], [21]. However, while the neurosphere model has been used for two decades, no standard protocol has been established to grow NSCs in this manner [11], [22], [23]. Moreover, the cellular and molecular mechanisms that underlie FGF-2/EGF maintenance of NSCs are not completely understood. Several studies of NSCs and cells isolated from other tissues, including embryonic stem cells (ESCs), suggest that FGF-2 fulfils a complex role, both when acting alone or in combination with other factors (e.g., EGF, BMP and IGF-I, among others). Indeed, FGF-2 directly or indirectly regulates the levels and postranscriptional state of a variety of molecular targets and it can affect self-renewal, cell survival, cell proliferation, adhesion and the suppression of terminal differentiation [11], [20], [24], [25], [26], [27], [28], [29], [30].

In the present study, NSCs isolated from the olfactory bulb were cultured and exposed to different FGF-2/EGF administration regimes in order to study the effects of these growth factors on cell proliferation, cell cycle progression, cell death and cell differentiation. Similarly, we used this paradigm to identify molecular mechanisms of FGF-2/EGF-mediated NSC survival and undifferentiation. Our findings provide an important basis for the standardization of NSC culture conditions, and they reveal novel molecular hallmarks of NSC death, survival, and the initiation of differentiation, including *alpha B Crystallin (Cryab)*, *phospholipase A2 group VII (platelet-activating factor acetylhydrolase*; *Pla2g7)*, *down syndrome cell adhesion molecule like (Dscaml1)*, *G-protein-coupled receptor* (*Gpr17), and N-myc downstream regulated gene2* (*Ndrg2).*

## Methods

### Ethical Statement

All animal care and handling was carried out in accordance with European Union guidelines (directive 86/609/EEC) and was approved by the Comisión de Bioética (Ethical Committee) of the Consejo Superior de Investigaciones Científicas (CSIC: certificates BFU2007-61230 issued on June 7, 2007 and BFU2010-1963 issued on September 24, 2010). All efforts were made to ameliorate the suffering of the animals.

### Neural Stem Cell Cultures

Adult olfactory bulb stem cells (aOBSCs) were prepared from 4- to 15-month-old CD1 mice or from adult C57Bl/6 mice following a protocol described previously [11]. As from a statistical point of view the results obtained with cultures from different mouse strains and adult mice of different ages were very similar, they were combined for analysis. Briefly, the OB were dissected out and cut into small pieces following removal of the meninges and blood vessels. The tissue was then digested with papain, gently disaggregated, and the resulting cell suspension was plated into 6-well plates containing Dulbecco’s modified Eagle medium (DMEM)/nutrient mixture F12 (F12), supplemented with insulin, apotransferrin, putrescine, progesterone, sodium selenite (N2: DMEM/F12-N2), or DMEMF12-B27 supplement in some experiments. The cells were passaged by mechanical procedures and were maintained until passage 3 with daily addition of both 20 ng/ml fibroblast growth factor-2 (FGF-2: Peprotech Cat No. 100-18B) and 20 ng/ml epidermal growth factor (EGF: Peprotech Cat No. AF-100-15) since the combination of both factors added at 20 ng/ml gives the maximum number of cells [11], [19]. Before their use, growth factors were reconstituted in 5 mM Tris [pH 7.6] and they were diluted in 0.01% bovine serum albumin to increase their stability. The cultures were then divided into three experimental groups, to which FGF-2 and EGF were added: (1) daily (control condition: Ctr); (2) every 2 days (C2 condition); and (3) every 4 days (C4 condition). Cells were maintained in these conditions for two additional passages before the effects of the frequency of growth factor addition was assayed on cell proliferation, cell death, cell differentiation and gene expression. For the most part, experiments were performed with cells before passage 12 when the majority of cells exhibit a stable karyotype [11].

Embryonic olfactory bulb stem cells (eOBSCs) were prepared from the OB of E13.5 CD1 mice as described previously [19], [31] and they were then expanded as neurospheres and plated as mentioned above for the experimental assays.

Cell proliferation assays were performed on floating neurospheres of eOBSCs grown for 4 days or aOBSCs grown for 7 days (density at plating: 5,000 cells/cm^2^), or on adherent cells cultured on polyornithine and fibronectin-coated glass coverslips (density at plating: 10,000 cells/cm^2^). Cells were exposed to a 30 minutes (neurosphere cultures) or 1 hour (adherent cultures) pulse of 5′-bromo-2-deoxyuridine (BrdU, 5 µM: Boehringer-Mannheim), a dose previously shown to have no toxic effects on NSC proliferation [11]. Neurospheres were then collected on growth factor-reduced matrigel (BD Biosciences) for 10 minutes and fixed with 4% paraformaldehyde (PFA) for 25 minutes. Adherent cultures were fixed directly.

For differentiation assays, cells were initially expanded as described, passaged and then cultured in DMEM/F12-N2 for 3 days in the absence of mitogens at a density of 100,000 cells/cm^2^ (short-term differentiation assay). Cells were then fixed with 4% PFA and immunostained.

Some aOBSC cultures were treated with 10 or 20 ng/ml vascular endothelial growth factor-C (VEGF-C RELIATech Cat. No. R20-014), 50 ng/ml fibroblast growth factor-8 (FGF-8: Peprotech Cat. No. 100-25) or 100 ng/ml sonic hedgehog (SHH: Peprotech Cat. No. 100-45B). These factors were chosen due to their expression in the OB, or that of their receptors, and based on previous reports describing their influence on cell proliferation and neurogenesis in telencephalic regions [32], [33], [34].

### Cell Cycle Analysis

The cell cycle of cultured cells growing under proliferation conditions was analysed at different time points. Cells were collected mechanically, washed with PBS and fixed overnight in 70% ethanol. After an additional wash, the cells were treated with RNAse A (Sigma) for 20 min at 37°C, incubated with propidium iodide (PI, final concentration 25 µg/ml) and then analyzed by flow cytometry (FACS Vantage, BD) to determine the proportion of cells in each phase of the cell cycle.

### Cell Death Assays

The FITC Annexin V Apoptosis Detection Kit I (BD, Cat. No. 556547) was used in conjunction with propidium iodide to detect cells undergoing early and late apoptosis in neurosphere cultures, according to the manufactureŕs instructions. Briefly, neurospheres were disaggregated at different time points, washed with cold PBS and incubated for 15 min at room temperature with Annexin V-FITC in binding buffer. Finally, propidium iodide (2.5 µg/ml) was added to the cells, which were subsequently analyzed by flow cytometry to measure the proportion of cells undergoing early and late apoptotic processes and cell death.

### Immunostaining of Cells in Neurosphere and Adherent Cultures

After treatment with 0.1% Triton X-100/10% NGS/PBS, cells were incubated overnight at 4°C with primary antibodies raised against: β-III-tubulin (TuJ1, rabbit antibody, 1∶2,000: Covance, Berkeley, CA, Cat. No. PRB-435P); GFAP (rabbit polyclonal, 1∶1,000: Dako, Glostrup, Denmark, Cat. No. Z0334); O4 (mouse monoclonal IgM, 1∶6 obtained from the culture media of O4-producing hybridoma cells, kindly provided by A. Rodríguez-Peña, CSIC, Madrid, Spain) or BrdU (mouse antibody, 1∶200: Becton Dickinson, San Jose, CA, Cat. No. 7580). The cells were then incubated with Alexa fluor 488 or 594 conjugated secondary antibodies (1∶500: Invitrogen) and finally with 4′,6-diamidino-2-phenylindole (DAPI: Vector Laboratories, Burlingame, CA) before mounting them in Mowiol solution (Calbiochem, San Diego, CA). Controls were performed to confirm the specificity of the primary and secondary antibodies.

### Cell Counts

To analyse neurosphere size, we used confocal images of optical planes taken every 3 µm along the thickness (Z axis) of the entire neurosphere and we estimated the volume using ImageJ software. After determining that the number of positive cells in the different planes was almost identical (4% difference between planes), the number of BrdU^+^, GFAP^+^ and TuJ1^+^-cells was counted in one plane only. The percentage of positive cells was expressed as the average ± SEM of cells relative to the total number of DAPI^+^-cells from 30 neurospheres in 2 experiments.

To determine the number of cells growing in adherent culture conditions that expressed a specific antigen, 10 random fields per coverslip were counted using a 40x objective and fluorescence filters. The number of TdT-mediated dUTP nick-end labelled (TUNEL)^+^ cells was counted in a similar manner. The percentage of cells positive for specific markers was calculated with respect to the total number of DAPI^+^-cells and the results expressed as the average (± SEM) number of cells in the 10 fields taken from 4-20 cultures in 2–6 experiments.

### Statistical Analysis

One way ANOVA was used to compare the percentage of cells between the three experimental groups (Ctr, C2 and C4) when no significant difference in variances was detected (Bartlett´s test), which was followed by *post hoc* analysis using Bonferronís test. In cases where variances differed, statistical analysis was performed using the Kruskal-Wallis test (a non-parametric method) followed by *post hoc* analysis using Dunńs multiple comparison test. To compare the percentage of cells between two experimental groups, we used a two-tailed Student’s *t*-test with Welch´s correction when the F-test indicated significant differences between the variances of both groups. All analyses were carried out with GraphPad Prism software.

### Microarray Methods, Gene Ontology and KEGG Analysis

#### RNA isolation and cDNA synthesis

Total RNA was extracted from aOBSC neurospheres after 7 days in culture using the Trizol reagent (Invitrogen) and purified with Qiagen RNeasy Mini Kit separation columns (Qiagen). The RNA integrity was confirmed using a Bioanalyzer in the Genomic Unit of the CNB (Centro Nacional de Biotecnología, Madrid, Spain). Next, to assess and compare overall gene expression profiles, cDNA was synthesized and hybridized to Affymetrix GeneChip Mouse Genome 430 2.0 arrays (Affymetrix, Santa Clara, CA, http://www.affymetrix.com), which contain a total of 45,101 transcripts.

#### Microarray data processing

Data were normalized using the GCRMA (GC content corrected Robust Multi-array Analysis) algorithm [35]. Data was post-processed and the graphics were designed using Matlab functions developed in-house. To compare gene expression between populations we used a fold-change threshold of 1 (in log_2_ scale). Hierarchical clustering of genes and samples was performed with the one minus correlation metric using the unweighted average distance (UPGMA: also known as the group average) linkage method [36].

#### Gene ontology enrichment analysis

Gene ontology (GO) terms were taken from the AMIGO database [37] and the significance of the GO terms for the differentially expressed genes was analyzed using an enrichment approach based on the hypergeometric distribution. All the sets of GO terms were back-propagated from the final term appearing in the gene annotation to the root term for each ontology. The significance (P-value) of GO term enrichment was calculated using the hypergeometric distribution. The multi-test effect influence was corrected by controlling the false discovery rate (FDR) using the Benjamini-Hochberg correction, with a significance level of α = 0.05.

As background set, we selected the genes, whose difference in expression between the samples with the lowest and the highest expression was at least 1 (in log_2_ scale).

#### Pairwise scatter plots

Pairwise scatter plots were used to compare the C2 and C4 conditions with the control. Each point in the pairwise scatter plot represents the expression of a transcript in the two samples. Using a fold-change of 1 in log2 scale: *N*-similar is the number of similarly expressed genes, *Per*-similar is the percentage of similarly expressed genes, *R* is the Fisher’s correlation coefficient, *N*-different is the number of differently expressed genes, and *Per*-different is the percentage of differentially expressed genes. The percentages were calculated with respect to the total number of transcripts in the microarray (45,101).

#### KEGG (Kyoto Encyclopedia of Genes and Genomes) pathway analysis

KEGG (Kyoto Encyclopedia of Genes and Genomes) pathways were downloaded from the KEGG database [38] and the significance of the KEGG pathways for the differentially expressed genes was analyzed using the same methodology as in the GO enrichment analysis (with the exception that there is no need for a back-propagation step).

### Real-Time Quantitative Reverse Transcription Polymerase Chain Reaction (RT-qPCR)

For this analysis, we considered probe sets whose expression varied in the microarray by two-fold or more in the C2 and C4 cultures as compared to control, and that had a FDR <0.01. The reverse transcription reaction was carried out with SuperScript III (Invitrogen) to synthesize cDNA from the mRNA obtained from aOBSC and eOBSC neurospheres that where cultured for 7 and 4 days, respectively. The sequences of primer pairs for qPCR were designed with Lasergene software and are listed in Table S1. All PCR cDNA products obtained were sequenced and correspond to the expected fragments. The optimal mRNA amounts for reverse transcription were determined using 1∶2 serial dilutions ranging from 1 µg to 125 ng. The dynamic range of amplification for each primer set was determined using serial dilutions of cDNA. Finally, a common optimal cDNA amount, which was within the dynamic range of all primer sets, was selected for all the PCR reactions. A real time qPCR analysis was performed in triplicate using Power SYBR Green (Applied Biosystems). The Ct value obtained for each target gene was normalized to the Ct value for *Gapdh* using the comparative C_T_ method. Then, gene expression changes in the C2 and C4 conditions were compared relative to the levels of gene expression obtained in the Ctr condition, using the C_T_ method [39] and were expressed as fold changes in log_2_ scale. The expression of *Fgf-2*, *Egf*, *Fgfr1-3*, and *Egfr* in aOBSCs and eOBSCs was also measured by RT-qPCR and the results were given as relative mRNA levels normalized to the Ct value for *Gapdh*.

## Results

It is well established that the addition of both human recombinant FGF-2 and EGF (FGF-2/EGF) is critical to maintain and expand NSC cultures as floating neurospheres [11], [15], [19], [21]. However, there is still no standard protocol to grow NSCs in this manner [11], [23]. In addition, the cellular and molecular mechanisms responsible for the survival and maintenance of the undifferentiated state of NSCs in response to FGF-2/EGF are not completely understood. Here, we analyzed the effects of three FGF-2/EGF administration regimens on cell proliferation, cell cycle progression, cell death, cell differentiation, as well as on global gene expression to identify molecular mechanisms of NSC survival and the initiation of differentiation. Thus, we maintained eOBSCs and aOBSCs in standard serum-free medium (DMEM/F12-N2) supplemented with FGF-2/EGF daily (Ctr condition), every 2 days (C2 condition) or every 4 days (C4 condition). It should be emphasized that when aOBSCs or eOBSCs are plated in the total deprivation of exogenous FGF-2/EGF, the cells stop diving and start to differentiate or die [11], [19].

These eOBSC and aOBSC populations were previously characterized as NSCs based on their capacity to self-renew and differentiate into neurons, astrocytes and oligodendrocytes under clonal conditions, as well as through the expression of NE and RG markers such as nestin, Sox2 and RC2, and the almost complete absence of differentiation markers when cultured in DMEM/F12/N2 medium with daily addition of FGF-2/EGF ([11], [19]; see also data herein). Moreover, these NSCs also generated neurons, astrocytes and oligodendrocytes when transplanted *in vivo*.

### Partial FGF-2/EGF Deprivation Affects Neurosphere Size and OBSC Undifferentiated State

**Figure 1:**
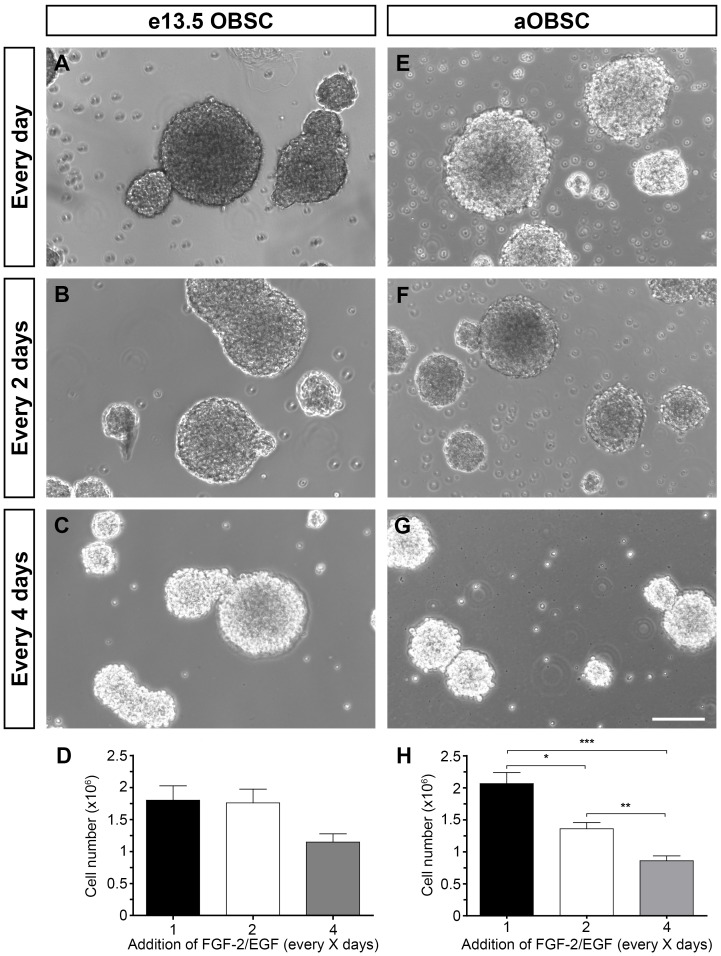
Growth of embryonic and adult OBSC neurospheres supplemented at different intervals with FGF-2 and EGF. Embryonic OBSCs (**A–D**) and adult OBSCs (prepared from 4-, 6-, 7-, 12- and 15-month old mice; **E–H**) were grown as neurospheres, and FGF-2 and EGF (FGF-2/EGF) were added daily, every 2 days or every 4 days. On the day of passage (day 4 for embryonic and day 7 for adult OBSCs), the neurospheres were mechanically dissociated and the cell number was determined using the Trypan blue dye exclusion method. Images show representative E13.5 (**A–C**) and adult (**E–G**) neurospheres. The bar graphs show the average number of eOBSCs (**D**) and aOBSCs (**H**) in each condition. The results represent the mean ± SEM from 24-35 passages from 6 different cell cultures per condition. Decreasing the frequency of FGF-2/EGF addition reduced cell number and neurosphere size. *P<0.05, **P<0.01 and ***P<0.001 (Kruskal-Wallis test followed by *post hoc* analysis using Dunn´s multiple comparison test). The 36% reduction in eOBSC number in the C4 versus de Ctr condition (**D**) was statistically significant when the two average means were compared using the Student´s t test (P<0.01). Scale bars (G) = 121.02 µm.

For each cell population, eOBSC and aOBSC, the experiments were performed in parallel using the same cell seeding densities and maintaining constant intervals between passages (4 days for eOBSCs; 7 days for aOBSCs). It should be noted that for partial FGF-2/EGF deprivation (C2/C4), eOBSCs remained 2/4 days without added FGF-2/EGF while aOBSCs were maintained for 1/3 days in these conditions before collecting the cells for the analyses. This was due to the fact that aOBSCs initially plated at a cell density of 5,000 cells/cm^2^ (our standard density for both eOBSC and aOBSC cultures) become excessively confluent after 8 DIV in the Ctr condition, which may *per se* trigger rapid cell death and a loss of cell viability (data not shown). Thus, we decided to analyze aOBSCs and eOBSCs after 7 and 4 DIV, respectively, maintaining the same initial cell density. The medium was not changed during these periods. Neurospheres formed from both eOBSCs and aOBSCs maintained with different intervals of FGF-2/EGF supplementation (Fig. 1). However, compared with the corresponding controls the size of the neurospheres appeared to decrease when FGF-2/EGF was added every 4 days to eOBSC cultures (Fig. 1 A, B and C), and every 2 and 4 days to aOBSC cultures (Fig. 1E, F and G). Moreover, on average the total number of cells counted in each passage in aOBSC cultures was significantly lower than in control cultures (Fig. 1H) when FGF-2/EGF were added every 2 (35%, P<0.05, ANOVA) or 4 days (58%, P<0.001, ANOVA). The total number of cells was 36% lower in eOBSC cultures supplied with FGF-2/EGF every 4 days than in controls (Fig. 1D). This reduction in cell number was statistically significant when the two average means (C4 versus the Ctr condition) were compared using the Student´s t test (P<0.01). Similar results were obtained when OBSCs were grown in DMEM/F12-B27. Accordingly, the data obtained in both DMEM/F12-N2 and DMEM/F12-B27 culture conditions were combined for analysis. However, in subsequent experiments the cells were grown exclusively in DMEM/F12-N2.

**Figure 2:**
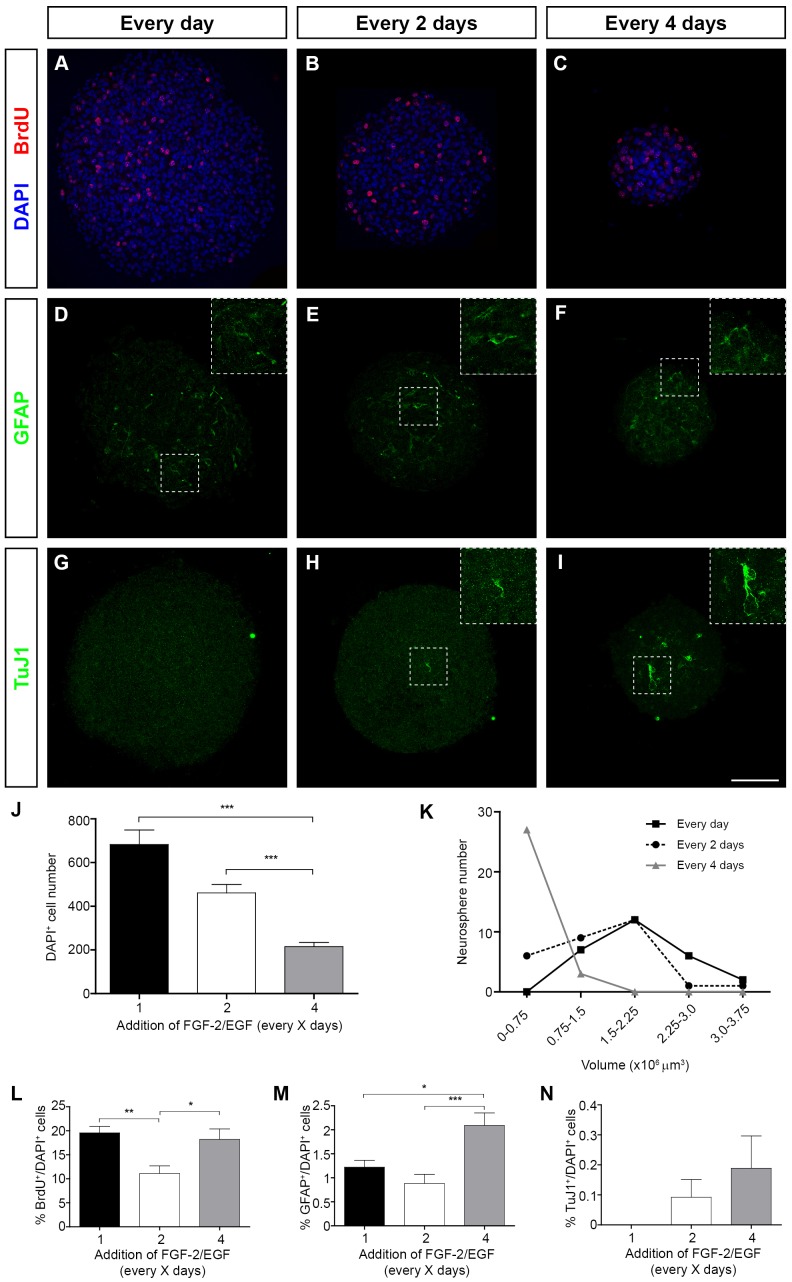
The effect of growth factor addition on neurosphere size, proliferation and the expression of cell differentiation markers in aOBSC neurospheres. The aOBSCs (prepared from 7- and 15-month old mice) were grown as neurospheres, to which FGF-2/EGF was added at different intervals. After 7 days in culture, BrdU (5 µM) was added for 30 minutes and the neurospheres were collected on matrigel, fixed and immunostained, and then stained with DAPI. Representative images of neurospheres grown in cultures supplemented with growth factors daily (**A, D, G**), every 2 days (**B, E, H**) and every 4 days (**C, F, I**), and immunostained with BrdU (**A–C**), GFAP (**D–F**) and TuJ1 (**G–I**). Bar graphs show the average number of DAPI^+^ cells per confocal plane of each neurosphere (**J**), and the percentage of BrdU^+^ (**L**), GFAP^+^ (**M**) and TuJ1^+^ cells (**N**). Line graphs show the distribution of neurosphere number versus volume (**K**). The results represent the mean ± SEM of 15–30 neurospheres from 2 different cell cultures. *P<0.05, **P<0.01 and ***P<0.001 (One way ANOVA followed by *post hoc* analysis using Bonferronís test). The 32% reduction in DAPI^+^ cell number in the C2 versus the Ctr condition (**J**) was statistically significant when the two average means were compared using the Student´s t test (P<0.01). Scale bars (I) = 77.51 µm and 39.0 µm (inserts).

The impact of the frequency of FGF-2/EGF addition on aOBSC growth was further evaluated in terms of cell proliferation, neurosphere size, cell death and the initiation of cell differentiation in neurospheres (Fig. 2). These were first incubated for 30 minutes with BrdU, collected in matrigel, fixed and immunostained. When compared with controls, the number of DAPI^+^ cells per neurosphere decreased in cultures treated with FGF-2/EGF every 2 days (32.6% reduction in the number of DAPI^+^ cells; non-statistically significant in the ANOVA test followed by post-hoc analysis; P<0.005 in the Student´s t test) and every 4 days (3.1-fold reduction; P<0.001, ANOVA) (Fig. 2J). We also observed a decrease in the number of DAPI^+^ cells in the C4 versus C2 condition (2-fold reduction; P<0.001, ANOVA). In C4 cultures, the majority of neurospheres were relatively small in volume (0–0.75×10^6^ µm^3^), and no neurospheres with a volume equal to or larger than 1.5×10^6^ µm^3^ were detected (Fig. 2K).

Compared with controls, the proportion of BrdU^+^ cells decreased by 43% (P<0.01) in C2 whereas non significant change was observed in C4 cultures (Fig. 2L). Decreasing the frequency of FGF-2/EGF application promoted the differentiation of aOBSCs to GFAP^+^ astrocytes (C4 condition, 70.9% increase in the proportion of GFAP^+^ cells versus controls; P<0.05) and TuJ1^+^ neurons (the proportion of TuJ1^+^ cells was 0.00%, 0.09%, and 0.19% in control, C2 and C4 cultures, respectively: Figure 2 M, N). All these results indicate that the addition of FGF-2/EGF at longer intervals promotes marked reductions in both the number of cells per neurosphere and the abundance of larger neurospheres. Moreover, these findings demonstrate that a small yet significant proportion of aOBSCs differentiate when growth factors are added every 4 days.

**Figure 3:**
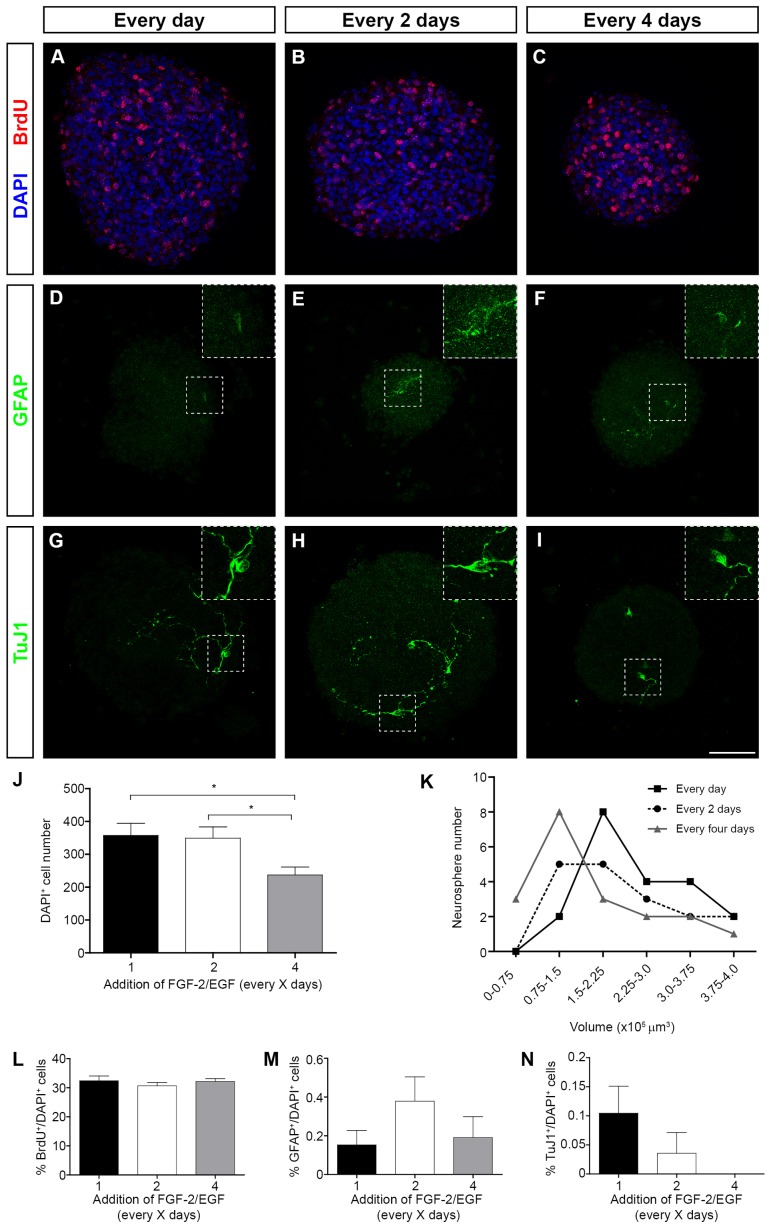
The effect of growth factor addition on neurosphere size, proliferation and expression of cell differentiation markers in eOBSC neurospheres. The eOBSCs were cultured and passaged as floating neurospheres as described in Fig. 1. After 4 days in culture, BrdU (5 µM) was added for 30 minutes and the neurospheres were collected on matrigel, immunostained and counterstained with DAPI. Images show the representative neurosphere growth in cultures to which factors were added daily (**A, D, G**), every 2 days (**B, E, H**) and every 4 days (**C, F, I**), and that were immunostained with BrdU (**A–C**), GFAP (**D–F**) and TuJ1 (**G–I**). Bar graphs show the number of DAPI^+^ cells (J), and the percentages of BrdU^+^ (**L**), GFAP^+^ (**M**) and TuJ1^+^ (**N**) cells. Line graphs show the distribution of neurosphere number versus volume (**K**). Results represent the mean ± SEM from 15–30 neurospheres in 2 experiments. *P<0.05 (One way ANOVA followed by *post hoc* analysis using Bonferronís test). Scale bars (I) = 77.51 µm, insert  = 39 µm.

We next performed the same analysis on eOBSC neurosphere cultures (Fig. 3). We observed a significant reduction (32–34%, P<0.05, ANOVA) in the number of DAPI^+^ cells per neurosphere in C4 cultures with respect to both C2 and Ctr cultures (Fig. 3 A–K) and there was a greater abundance of smaller neurospheres (ranging between 0–0.75×10^5^ and 0.75–1.5×10^5^ µm^3^: Fig. 3 K). The proportion of BrdU^+^, GFAP^+^ and TuJ1^+^ cells in eOBSC neurospheres did not differ significantly between the three conditions (Fig. 3 A–I, L–N). These results show that eOBSC growth is altered when exogenous FGF-2/EGF is added only every 4 days, an effect best witnessed by the significant reduction in neurosphere size.

FGF-2 and EGF are the extracellular growth factors most commonly used to expand NSCs in culture. Nevertheless, we also investigated whether the addition of VEGF-C, FGF-8 or SHH to aOBSC cultures (which are more dependent on exogenous FGF-2/EGF than eOBSCs), in conjunction with FGF-2/EGF, influenced NSC proliferation and their differentiation. The proportion of cells that incorporated BrdU relative to the total number of DAPI^+^ cells was similar in all conditions (10–15%: Fig. S1 A-D, G), as was the proportion of differentiating neurons (14–15%: Fig. S1 E, F, H). Accordingly, only the effects of FGF-2/EGF on cell cycle progression, cell death and differentiation were analyzed further in both eOBSCs and aOBSCs.

### Effect of Partial FGF-2/EGF Deprivation on OBSC Survival and Cell Cycle Parameters

**Figure 4:**
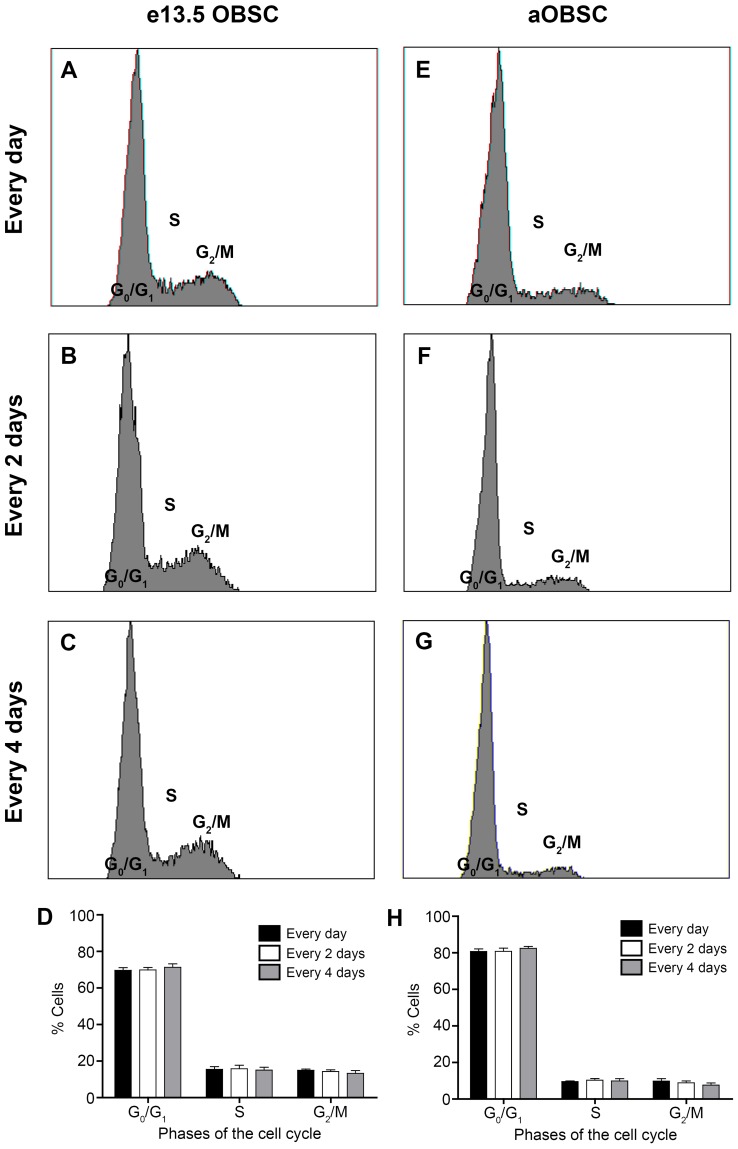
The influence of the frequency of FGF-2/EGF addition on the OBSC cell cycle. Embryonic OBSCs (**A–D**) and adult OBSCs (prepared from 7- and 15-month old mice; **E–H**) were grown as neurospheres as described in Fig. 1. After mechanical dissociation on days 4 (eOBSC) or 7 (aOBSCs), cells were fixed with ethanol, stained with PI and analyzed by flow cytometry. Graphs show the representative cell cycle profiles for eOBSC (**A–C**) and aOBSC (**E–G**) cultures, to which FGF-2/EGF was added daily (**A, E**), every 2 days (**B, F**) or every 4 days (**C, G**). The graphs show the percentage of cells in the different phases of the cell cycle (**D**, eOBSCs; **H**, aOBSCs). Results represent the mean ± SEM from 4 passages from 2 different cell cultures per condition. Under these conditions, the addition of growth factors at different intervals had no significant effect on the cell cycle parameters tested. However, aOBSC cultures contained more cells in G_0_/G_1_ (P<0.001) and fewer cells in S and G_2_/M (P<0.001) as compared with eOBSCs (two-tailed Student’s *t*-test with Welch´s correction).

The decrease in the total number of cells observed in cultures supplemented with FGF-2/EGF every 2 or 4 days (Fig. 1) and in the neurospheres size (Figs. 2 and 3), may be due to changes in cell cycle parameters and/or increased cell death. To test these hypotheses in depth, we first used flow cytometry to analyze the proportion of cells in each phase of the cell cycle (Fig. 4). In both eOBSC and aOBSC cultures analysed at 4 DIV and 7 DIV, respectively, the percentages of cells in the G_0_/G_1_, S and G_2_/M phases were similar and apparently independent of the frequency of FGF-2/EGF addition (Fig. 4 D, H). To further examine whether the changes in cell cycle could have occurred at earlier time points after partial FGF-2/EGF deprivation, the proportion of cells in each cell cycle phase was determined in eOBSC cultures at 2, 3 or 4 DIV, and in aOBSC cultures at 5, 6 or 7 DIV growing in both the C4 and Ctr conditions. No consistent changes were detected in eOBSC cultures (Fig. S2 left, average data from 2 cultures) and indeed, the proportion of eOBSCs in G_2_/M after 2 DIV was 57% greater in the C4 than in the control condition (9.6±2.1% *vs* with 6.1±0.00005), whereas at 3 DIV the proportion of eOBSCs in G_2_/M was 38% lower in the C4 than in the controls (5.2±0.2% *vs* with 8.4±1.3). Conversely, the percentages of aOBSCs in the G_0_/G_1_ phase were 5–12% higher in C4 than in controls (Fig. S2 right), whereas the percentages of cells in the S and G_2_/M phases were 4–21% and 8–48% lower in C4 than in the controls, respectively, although these changes did not reach statistical significance in the analysis performed (for instance, P = 0.33 for G_2_/M data at 7 DIV). However, compared to eOBSCs, the results obtained in aOBSCs were more suggestive that partial FGF-2/EGF deprivation possibly affected the cell cycle.

In aOBSC cultures (7 DIV) the average proportion of cells in the G_0_/G_1_ phase (81.3%) was significantly higher than that in eOBSC cultures (4 DIV; 70.3%: P<0.0001, Student’s *t*-test; Fig 4 D and H). Consequently, the percentage of cells in the S phase and in G_2_/M phase was significantly lower (P<0.0001, Student’s *t*-test) in aOBSC cultures (9.9% and 8.8%, respectively) than in eOBSC cultures (15.4% and 14.2%, respectively). These findings suggest a larger proportion of cells are quiescent and/or retained in the G_1_ phase in aOBSC cultures over time.

**Figure 5:**
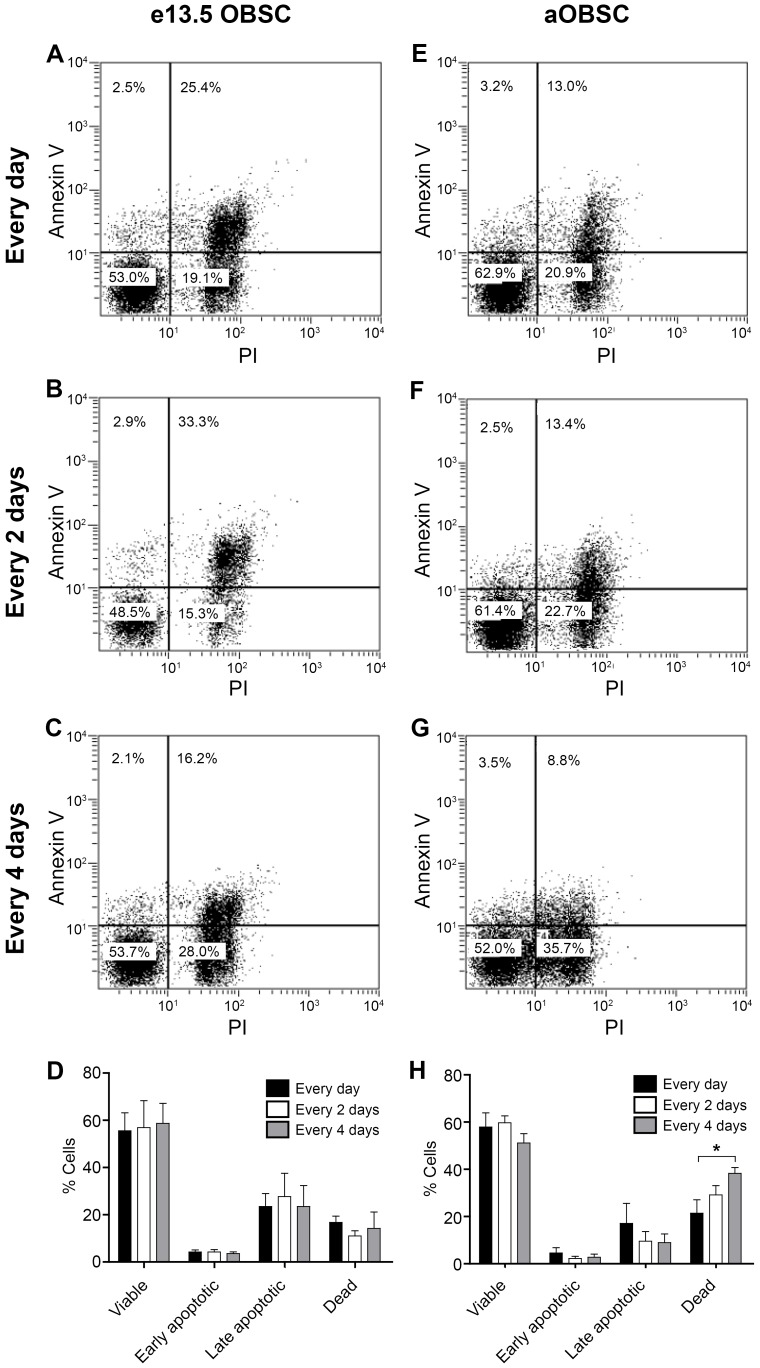
Decrease in the frequency of FGF-2/EGF addition increases cell death in aOBSCs. Embryonic (**A–D**) and adult (prepared from 7- and 15-month old mice; **E–H**) OBSCs were grown as neurospheres as described in Fig. 1. On days 4 (eOBSCs) or 7 (aOBSCs) the neurospheres were dissociated mechanically, and the cells were stained with PI and annexin V before they were analyzed by flow cytometry. Dot-plots show the percentage of viable, early and late apoptotic and dead cells in the cultures to which growth factors were added daily (**A, E**), every 2 days (**B, F**) or every 4 days (**C, G**). Graphs show the percentage of cells in each group (**D**, E13.5 OBSCs; **H**, aOBSCs). The results represent the mean ± SEM from 3–5 passages from 2 different cell cultures per condition. A decrease in the frequency of growth factor addition promoted significant cell death in aOBSCs but not in eOBSCs. *P<0.05 (One way ANOVA followed by *post hoc* analysis using Bonferronís test). PI = Propidium iodide.

After 4 DIV in eOBSCs and 7 DIV in aOBSCs, we next analyzed the effects of FGF-2/EGF supplementation at different intervals on the number of cells undergoing apoptotic processes and cell death (Fig. 5). At the aforementioned time points, the proportion of viable cells (annexin V^-^ and PI^-^) decreased slightly in the C4 condition (12%; non statistically significant). In addition, we observed more dead cells (PI^+^) in aOBSC cultures treated with growth factors every 2 or 4 days than in control cultures. Indeed, with respect to the control aOBSCs there were 36% more dead cells in the C2 condition (non statistically significant) and 78.9% more dead cells in the C4 condition (P<0.05, ANOVA). To test whether the changes in cell death could have occurred at earlier time points after partial FGF-2/EGF deprivation, the proportion of viable, apoptotic and dead cells was determined at 1, 2, 3 or 4 DIV in eOBSC cultures, and at 5, 6 or 7 DIV in aOBSC cultures growing in the C4 and Control conditions. There were no apparent changes in eOBSC cultures (Fig. S3 left, average data from 2 cultures) whereas the percentage of viable cells in partial FGF-2/EGF starved aOBSC cultures decreased (5–27.6%; P<0.05 and P<0.001 for 6 and 7 DIV, respectively, average data from 2 cultures), and the percentages in apoptotic cells increased at all the time points analyzed (early apoptotic, 32.1–39.4%; late apoptotic, 37.2–85.5%; P = 0.07 for late apoptotic cells at 7 DIV). The proportion of dead cells in aOBSC cultures also augmented (1.9–3.1-fold; P = 0.12 at 7 DIV: Fig. S3, right). These data indicate that partial deprivation of FGF-2/EGF causes a progressive loss in cell viability in aOBSC cultures, possibly through apoptotic and necrotic mechanisms, and that aOBSCs are more dependent on FGF-2/EGF for their survival than eOBSCs. It should be noted that even though eOBSCs were without additional FGF-2/EGF on days 2/4 before analysis in conditions C2/C4, and aOBSCs were without additional FGF-2/EGF on days 1/3, the aOBSCs were more prone to undergo cell death, particularly in the C4 condition. Moreover, reductions in neurosphere volume and total cell number, and possible changes in cell cycle parameters were always more evident in aOBSC than in eOBSC cultures.

### Partial FGF-2/EGF Deprivation does not Significantly affect OBSC Multilineage Differentiation in Population Assays

We next investigated how FGF-2/EGF application at different intervals in the proliferative phase (in the figures called “growth factor pretreatment”) affected the generation of neurons, astrocytes and oligodendrocytes in short-term differentiation assays (3 days) of aOBSC (Fig. S4) and eOBSC (Fig. S5) cultures. We found no significant differences between the distinct treatment groups, although we observed a slight decrease (20–24%; ANOVA P = 0.3618) in the percentage of TuJ1^+^ neurons generated from aOBSCs in the C4 and C2 cultures, when compared with controls (Fig. S4 J). The absolute numbers of TuJ1^+^ neurons, GFAP^+^ astrocytes and O4^+^ oligodendrocytes in the differentiating aOBSC and eOBSC cultures were similar in all three culture conditions. These results indicate that OBSC multilineage differentiation (in the total absence of factors) is not affected by the frequency of FGF-2/EGF application to neurospheres growing under population conditions.

### Expression of *Fgf-2*, *Egf* and their Receptors in aOBSCs and eOBSCs

**Figure 6:**
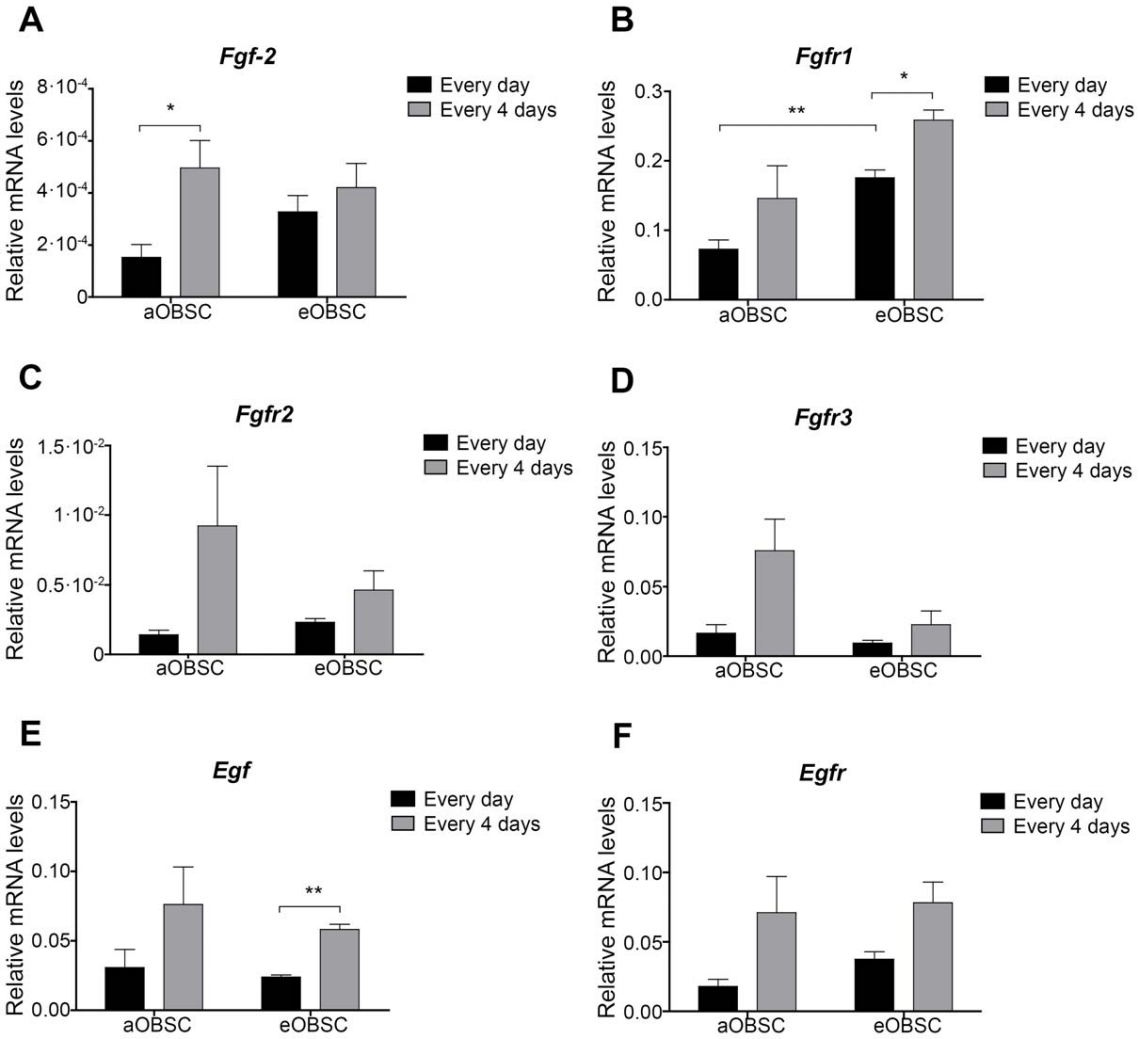
Expression of *Fgf-2*, *Egf* and their receptors in aOBSCs and eOBSCs. Embryonic OBSCs and adult OBSCs (prepared from 7- and 15-month old mice) were grown as neurospheres as described in Fig. 1. After mechanical dissociation on days 4 (eOBSC) or 7 (aOBSCs), mRNA was extracted from cells supplied with FGF-2/EGF every day (Ctr) and every 4 days (C4), and analyzed using real time RT-qPCR. The graphs show the relative mRNA levels of *Fgf-2* (**A**), *Fgfr1* (**B**), *Fgfr2* (**C**), *Fgfr3* (**D**), *Egf* (**E**) and *Egfr* (**F**) in Ctr and C4 cultures. aOBSCs partially starved of exogenous FGF-2/EGF expressed higher levels of endogenous *Fgf-2* than controls. The results represent the mean ± SEM of 3 experiments. *P<0.05, **P<0.01 (two-tailed Student’s *t*-test).

To investigate whether the different levels of dependence to FGF-2/EGF addition of aOBSC and eOBSC cultures for their survival, could be due to changes in the endogenous expression of the growth factors and/or their receptors between both OBSC populations, we analyzed the expression of: *Fgf-2*, its receptors more abundantly detected in the brain (*Fgfr1*, *Fgfr2*, *Fgfr3*; [40]), *Egf* and *Egfr* by RT-qPCR (Fig. 6). As shown in Fig. 6B, eOBSC neurospheres maintained with daily addition of FGF-2/EGF, expressed higher levels of *Fgfr1* compared with aOBSC growing under the same conditions (P<0.01, Student’s *t*-test). Moreover, partial FGF-2/EGF deprivation produced statistically significant upregulation in the expression of *Fgf-2* in aOBSCs (Fig. 6A) and of *Fgfr1* (Fig. 6B) and *Egf* in eOBSCs (Fig. 6 E). Although the expression of *Fgfr2* (Fig. 6C), *Fgfr3* (Fig. 6D), *Egf* (Fig. 6E), and *Egfr* (Fig. 6F) appeared to be upregulated in the C4 compared to the Ctr condition, the increases in relative mRNA levels did not reach statistical significance in these assays. Notably, the relative mRNA levels of *Fgf-2* in the cells were 20–155 fold lower than those of *Egf* (with comparable amplification efficiencies close to 100% for both transcripts), suggesting that the endogenous FGF-2 signaling pathway could be a more limiting process than that of EGF for the regulation of OBSC survival and maintenance.

### Molecular Mechanisms Underlying NSC Survival, and the Initiation of Differentiation

**Figure 7:**
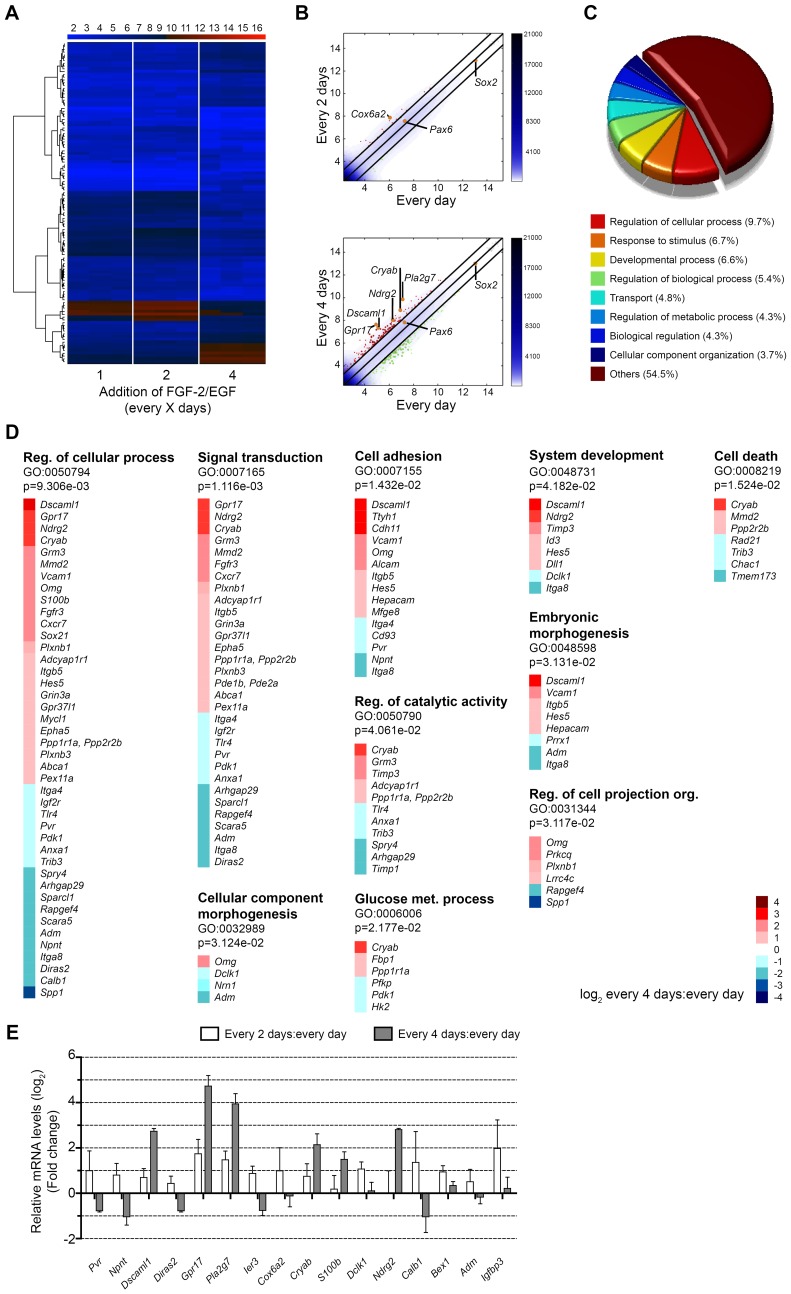
RNA expression in aOBSCs reveals previously undescribed genes involved in NSC death, survival and the initiation of differentiation. aOBSCs (prepared from 7- and 15-month old mice) were cultured and passaged as floating neurospheres as described in Fig. 1. The relative mRNA levels were analyzed using microarray and real time RT-qPCR techniques. (**A**) The heat map was generated using probes with a range of variation between samples of at least 2 and a FDR <0.01. The color bar at the top represents the gene expression in a log_2_ scale: the higher the gene expression level, the redder the color. (**B**) Pairwise scatter plots of the C2 (top) and C4 (lower) conditions relative to the Ctr condition. The black lines define the boundaries of the two-fold changes in gene expression between paired samples. Genes up-regulated in ordinate versus abscissas samples are shown in red circles, while down-regulated genes are shown in green. Orange dots indicate the positions of some key genes, including markers of NSCs (*Sox2* and *Pax6*) and of upregulated genes (*Cox6a2, Pla2g7, Cryab, Ndrg2, Dscaml1 and Gpr17*). The color bar to the right indicates the scattering density, whereby the higher the scattering density, the darker the blue color. (**C**) The pie chart shows the frequency of GO terms in the “biological process” category. (**D**) GO enrichment analysis revealed up to 10 GO terms that were statistically enriched in C4 cultures when compared with the controls. (**E**) The graph shows the relative changes in the mRNA levels of selected transcripts expressed in C2 and C4 cultures when compared with controls, and as measured by real time RT-qPCR. The aOBSCs supplemented every 4 days with growth factors exhibited alterations in genes involved in cell death and survival (*Cryab*), lipid catabolic processes (*Pla2g7*), cell adhesion (*Dscaml1*), cell differentiation (*Dscaml1*, *Gpr17*, *S100b, Ndrg2*) and signal transduction (*Gpr17*, *Ndrg2).* The results represent the mean ± SEM of 3 experiments.

The marked reduction in the survival of aOBSCs (and to a lesser extent eOBSCs) and the increased tendency of aOBSCs to differentiate in the proliferative phase following partial FGF-2/EGF deprivation prompted us to investigate the potential changes in gene expression and signaling pathways that may underlie these effects. Genome-wide mRNA profiling by microarray analysis of aOBSCs grown under each condition revealed differential expression (*i.e.,* up or downregulation by at least one-fold in log2 scale) of 38 and 312 genes in the C2 and C4 condition, respectively. In the C4 condition, 180 genes were upregulated and 132 down-regulated with respect to the controls. These results are illustrated in the heat map of the global analysis (Fig. 7 A), which shows a marked difference between the C4 and control samples, while a comparison of the C2 and Ctr groups revealed small differences. The hierarchical clustering analysis confirmed the differences between C4, C2 and control cultures (Fig. S6). The shorter the horizontal link that connects two branches, the closer the populations represented by the branches. In the pairwise scatter plots (Fig. 7B), the position of some key genes is represented by orange dots. The expression of *Sox2, Pax6* and *Olig2* (not shown), which are considered markers of neural stem and progenitor cells [11], [36], [41], [42], [43], was similar in the three conditions studied. By contrast, other genes were differentially expressed (upregulated) in C4 versus control cultures, including the *phospholipase A2 group VII (platelet-activating factor acetylhydrolase*; *Pla2g7)*, *alpha B Crystallin (Cryab)*, *N-myc downstream regulated gene2* (*Ndrg2)*, *down syndrome cell adhesion molecule like (Dscaml1)* and *G-protein-coupled receptor* (*Gpr17)* (see below). We performed a Gene Ontology (GO) analysis of genes differentially expressed in C4 versus control cultures (Fig. 7 C-D; Fig. S9), dividing each GO analysis into two parts. When we searched the terms most often associated with a particular gene ontology category (molecular function, biological process or cellular component), in the “biological process” category the frequency of GO terms was highest for genes associated with the “regulation of cellular process” term, followed by “response to stimulus” and “developmental process” (see pie chart in Fig. 7 C).

The GO significance analysis of enrichment revealed 10 significant GO terms associated with the genes differentially expressed in the C4 versus the Ctr group: “regulation of cellular process”; “signal transduction”; “cell adhesion”; “system development”; “cell death”; “regulation of catalytic activity”; “embryonic morphogenesis”; “cellular component morphogenesis”; “glucose metabolic process”; and “regulation of cell projection organization”. Among the upregulated genes in the C4 condition associated with such GO terms, we found *Cryab, Dscaml1, Ndrg2* and *Gpr17*, among others (Fig. 7 D). These genes have been implicated in cell death and survival (*Cryab*) [44], [45], [46], cell adhesion and neuronal differentiation (*Dscaml1*) [47], [48], signal transduction and both, neuronal and glia differentiation (*Gpr17*, *Ndrg2*) [49], [50], [51], [52], [53], [54], [55]. We also found genes, such as *Spry4* and *Adm* that had expression levels markedly lower in C4 conditions than in control cultures and that may be important for the control of cell proliferation and FGF-2 mediated signaling [28], [40], [56], [57]. Similarly, the expression of *Calb1* (*Calbindin*) was downregulated in the C4 condition. Calbindin is involved in neuronal survival and differentiation in response to neurotrophins and FGF-2 [58], [59], [60], [61]. In contrast, the levels of *S100b* (a Ca-binding protein) and *Fgfr3* were increased in the C4 condition, two genes expressed in cells of the astrocyte lineage [62], [63].

When the GO term enrichment analysis was performed for genes that were upregulated in the C4 versus Ctr conditions, we found as significantly enriched GO term the “lipid catabolic process”, which includes the highly upregulated gene *Pla2g7,* involved in the breakdown of toxic oxidized phospholipid products (Fig. S7A; Fig. 7) [64], [65]. In the analysis of downregulated genes in C4 cultures we found *Spry4 and Adm*. These genes are involved in cell proliferation [28], [56], [57] and were annotated within the significantly enriched GO terms, “regulation of kinase activity” and “response to peptide hormone stimulus” (Fig. S7 B).

**Figure 8:**
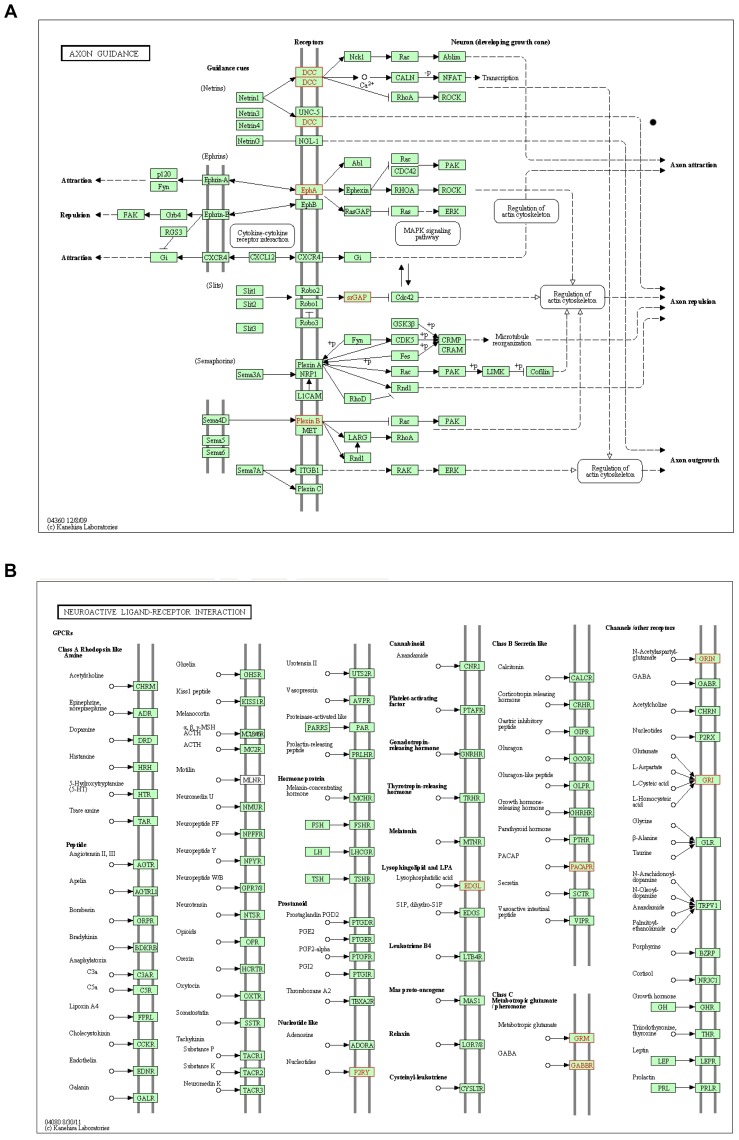
Maps of the KEGG significantly enriched pathways associated with genes upregulated in C4 versus control cultures. The KEGG pathway enrichment analysis revealed two significantly enriched pathways with the following maps. (**A**) axon guidance (*p* = 6.274e-03; *Dcc, Eph5a, Plxnb1, Plxnb3 and Srgap3*); and (**B**) neuroactive ligand-receptor interaction (*p* = 1.834e−02; *Adcyap1r1, Gabbr1, Grin3a, Grm3 and Lpar4*). In each map the upregulated genes are depicted in red.

To validate the differential expression of genes that were up- or down-regulated in C2 and C4 versus control cultures and that were associated with enriched GO terms, RNA extracts were analyzed by quantitative RT-PCR (RT-qPCR; Fig. 7E) using the primers listed in Table S1. We also measured the expression of other genes (*Ier3, Cox6a2, Bex1 and Igfbp3*) that were differentially expressed in the global microarray analysis by at least two fold but that were not associated with enriched GO terms. This analysis was also extended to RNA extracts from eOBSCs grown in all three FGF-2/EGF administration regimes (Fig. S8). A strong correlation was observed between the microarray and RT-qPCR results (coefficient of determination, R^2^ = 0.8477; Fig. S9). Indeed, expression of *Dscaml1*, *Gpr17*, *Pla2g7*, *Cryab*, *S100b* and *Ndrg2* was more than one-fold greater (in log_2_ scale) in C4 versus Ctr cultures, while *Gpr17*, Pla2g7, *Calb1* and *Igfbp3* were also upregulated in C2 cultures when compared with controls (Figure 7 E). Furthermore, the expression levels of *Dscaml1*, *Gpr17*, *Pla2g7* and *Ndgr2* were more than one-fold higher in C4 and C2 eOBSC cultures with respect to the corresponding controls (Fig. S8). Of the downregulated genes analyzed, only the expression of *Npnt* and *Calb1* was lower in C4 aOBSC cultures than in the controls (Fig. 7 E), although the expression of *Ier3* was also reduced in C4 eOBSC cultures by one-fold (Fig. S8). A Kyoto Encyclopedia of Genes and Genomes (KEGG) pathway enrichment analysis revealed that the “Axon guidance” and “Neuroactive ligand receptor interaction” pathways were significantly enriched in C4 cultures (Fig. 8). Some of the genes in the KEGG analysis were the same as those upregulated in the C4 group in the global profiling analysis, or they belonged to the same gene family. These included *Dcc*, *Plxnb1* and *EphA,* which are involved in the regulation of axon outgrowth and repulsion [48], [66], and *Grm3* and *Grin3a* that play a role in metabotropic glutamate signaling (Fig. 7 D) [67], [68].

Taken together, the results of our genome-wide profiling analysis reveal that reducing the frequency of FGF-2/EGF addition to OBSCs triggers significant changes in the expression of genes implicated in NSC death, protection, as well as in neuronal and glia differentiation.

## Discussion

The combined addition of exogenous FGF-2 and EGF (FGF-2/EGF) is critical to maintain and expand NSC cultures as floating neurospheres [11], [15], [19], [21]. However, while the neurosphere model has been used for two decades, important parameters that may affect the growth of NSCs cultured in this way are yet to be fully standardized, including the periodicity at which FGF-2/EGF should be administered [11], [23]. Moreover, the cellular and molecular mechanisms responsible for the maintenance of NSCs in response to FGF-2/EGF may be complex and they are not completely understood. We previously reported elsewhere that the addition of both FGF-2 and EGF were necessary to yield the maximum number of proliferating cells [11], [19]. Here we used a paradigm of partial FGF-2/EGF deprivation in NSC cultures established from the embryonic and adult OB (eOBSCs and aOBSCs) to study the consequences of such deprivation on cell proliferation, cell cycle progression, cell death and cell differentiation, as well as on the molecular mechanisms acting downstream of these growth factors.

The results reported here indicate that a daily supply of exogenous FGF-2/EGF is critical to maintain the survival and undifferentiated state of OBSCs growing as neurospheres (especially that of aOBSCs), whereas their proliferation appears to be less dependent on a daily supply of these growth factors. Furthermore, we present evidence that the partial deprivation of FGF-2/EGF to cultures induces cell death, cell protection and molecular events associated with cell differentiation. Indeed, our global transcriptome analysis revealed significant changes in 312 genes when FGF-2/EGF were added to the cultures every 4 days (C4 condition), and a GO enrichment analysis of such differentially expressed genes revealed significant differences for 10 GO terms in these conditions. Moreover, the KEGG enrichment analysis showed that two signaling pathways of differentiation were significantly enriched in C4 cultures. These results demonstrate that decreasing the frequency at which FGF-2/EGF is added to aOBSC cultures produces widespread changes at the transcriptome level.

### Both Cell Death and Cell Protection Mechanisms are Induced in Response to Partial Deprivation of FGF-2/EGF

As mentioned above, when FGF-2/EGF were added less often (especially at 4-day intervals), an increase in OBSC death was observed, and this could be the main cellular mechanism underlying the significant reduction in neurosphere volume and the total number of cells in the spheres derived from both aOBSC and eOBSC cultures. In aOBSC cultures (C4 condition), the proportion of cells in the G_0_/G_1_ phase of the cell cycle was slightly higher, and those in the S and G_2_/M phases slightly decreased over time. Along with the significant reduction of BrdU^+^ cells in the C2 cultures, these data suggest that aOBSC proliferation could be affected by partial FGF-2/EGF deprivation, although to a lesser extent than cell survival. By contrast, no consistent changes were detected in cell cycle parameters and BrdU incorporation in eOBSC cultures following partial FGF-2/EGF deprivation. This was an unexpected result as FGF-2 is known to be necessary for progenitor cell proliferation in the developing brain *in vivo* [40], [69], [70], [71], and both FGF-2 and EGF stimulate the proliferation of NSCs and progenitors isolated from the embryonic brain [24], [25], [29], [58], [72], [73], [74], [75]. One possible explanation for the distinct behavior of the two populations could be related to the significantly higher levels of *Fgfr1* (2.4-fold, p<0.001), and to the tendency of *Fgf-2* expression to be higher (2-fold, P = 0.09) in eOBSCs than in aOBSCs. Together, these changes could augment the activity of the FGF-2/FGFR1 signaling pathway in eOBSCs, diminishing the dependence of these cells on exogenous FGF-2. Accordingly, of the two OBSC populations, eOBSCs were less dependent on daily addition of FGF-2/EGF for survival and proliferation. However, a conjunction of minor changes in cell death and in cell cycle progression not detected in our analyses could contribute to the reduction in the total cell number and neurosphere size in the eOBSCs growing under the C4 conditions.

Consistent with the stronger impact of partial FGF-2/EGF deprivation on cell death than on cell proliferation, the GO term “cell division” was not significantly enriched when the FGF-2/EGF administration regime was changed. However, the microarray analysis revealed significant downregulation of the expression of *Spry4* and *Adm* transcripts in the C4 condition. Although these changes could be important for the control of cell proliferation as Spry4 regulates the FGF-2 signaling pathway and the lack of adrenomedullin alters cell proliferation in the olfactory bulb [28], [56], [57], no significant changes between the three conditions (Ctr, C2 and C4) were observed when the expression of *Adm* was determined by qPCR. Together, our findings suggest that the maintenance of OBSC survival requires a more continuous supply of FGF-2/EGF than is necessary for cell proliferation, possibly because the proliferative signaling activated by FGF-2/EGF lasts longer than that necessary to maintain cell survival, and/or because proliferation is activated by smaller doses of the growth factors. Nonetheless, as previously mentioned, it should be emphasized that when aOBSCs or eOBSCs are plated in the total deprivation of exogenous FGF-2/EGF, the cells stop diving and start to differentiate or die [11], [19].

It could be argued that requiring a constant supply of FGF-2/EGF for NSC survival is solely an *in vitro* adaptation to prevent the cell death caused by the culture conditions, such as maintaining NSCs in a hyperoxic atmosphere and their passage outside the *in vivo* niches in which their survival is likely to be controlled tightly. Although this hypothesis cannot be completely ruled out, it is worth mentioning that together with differentiation, cell death appears to be a natural fate of NSCs and progenitors both during CNS development [25], [76] and adult neurogenesis [77]. Furthermore, physiological aging, brain lesion and neurodegenerative diseases may negatively affect the survival of NSCs *in vivo*, implicating events related to death and inflammation [78], [79]. Accordingly, our findings might be relevant to shed light on the mechanisms that lead to the decrease in NSC number under physiological and pathological conditions, as well as to determine the responses of the cells as protective feedback mechanisms *in vivo*.

Our data show, for the first time, that *Cryab* is upregulated in aOBCs growing under conditions of FGF-2/EGF partial deprivation. *Cryab* codes for a chaperone reported to have anti-apoptotic, neuroprotective and anti-inflammatory functions [44], [45], [46]. Similarly, both aOBSCs and eOBSCs upregulate the *Pla2g7* that is involved in the breakdown of toxic oxidized phospholipid products that provoke apoptosis and necrosis [64], [65]. *Ndrg2* and *Gpr17* were also upregulated in both populations, which in addition to their possible implication in neuronal, astrocyte and oligodendrocyte differentiation, may also be stress-associated proteins and sensors of brain damage [49], [50], [51], [52], [53], [54]. Moreover, the significant upregulation of endogenous *Fgf-2* in aOBSCs and of *Egf* in eOBSCs could be part of the OBSĆs feedback mechanism to promote their survival and maintenance of the undifferentiated state when exogenous FGF-2/EGF are added less frequently. Conversely, the expression of *Calb1* was downregulated in the C4 condition. Calbindin is involved in neuronal survival and differentiation in response to neurotrophins and FGF-2, probably by buffering calcium [58], [59], [60], [61]. Moreover, calbindin and Ca-dependent signaling pathways have recently been implicated in adult NSC maintenance [63].

The potential relevance of our findings *in vivo* is further supported by the reported expression of the aforementioned gene products in the adult OB. *Cryab* mRNA is abundantly expressed in all the layers of the adult OB but only weakly in its subependymal zone (SEZ). *Pla2g7* and *Ndrg2* mRNA transcripts are distributed throughout the OB, including the SEZ, while *Gpr17* mRNA has a more scattered distribution in the OB, mainly in the granule cell layer (GCL) and the external plexiform layer (EPL: see The Allen Brain Atlas). Finally, calbindin is expressed by periglomerular neurons [80].

### Decreasing the Periodicity of FGF-2/EGF Administration Drives Differentiation in Cultured NSC: Molecular Mechanisms

Partial deprivation of FGF-2/EGF induced an increase in aOBSC differentiation during the proliferative phase, as reflected by the significant rise in the proportion of GFAP^+^ astrocytes and the appearance of TuJ1^+^ neurons in aOBSC neurosphere cultures supplied with FGF-2/EGF every 4 days. Indeed, the initiation of differentiation in aOBSCs was confirmed by our transcriptome analysis followed by RT-qPCR, which revealed a significant increase in the expression of genes related to neuronal, astrocyte and oligodendrocyte differentiation, such as *Dscaml1, S100b, Ndrg2* and *Gpr17* [47], [48], [49], [50], [51], [52], [53], [54], [55]. The upregulation of *Dscaml1*, *Gpr17*, and *Ndgr2* was also confirmed in eOBSCs.

While the upregulation (observed in the microarray analysis) of five more genes also involved in neuronal and glial differentiation as well as in neuronal migration (*Ttyh1*, *Vcam1*, *Omg*, *Fgfr3, and Cdh11*) [62], [63], [81], [82], [83], [84], [85] was not measured by qPCR, it cannot be ruled out that their higher expression in the C4 condition might also be relevant based on the high correlation between our microarray and RT-qPCR data. The proteins codified by *Dscaml1* and *Vcam1* fulfill roles in cell adhesion, repulsion and the formation of neuronal networks [82], [86], which supports the previously proposed role of adhesion as a critical *in vivo* and *in vitro* regulator of the maintenance and differentiation of NSCs, and of other stem cell populations [26], [63], [87], [88]. Indeed, *Dscaml1* mRNA is strongly expressed in the OB, particularly in layers that contain many differentiated neurons (i.e., the mitral layer -ML-, the superficial GCL and the glomerular layer -GL-) [47] (The Allen Brain Atlas). Our KEGG analysis further supports the critical role of a continuous supply of FGF-2/EGF in maintaining the undifferentiated state of OBSCs. Thus, genes that were upregulated in cultures partially starved of growth factors influence signaling pathways that control axon guidance, such as *Dcc*, *Plxnb1* and *Eph* [48], [66], and metabotropic glutamate signaling (*Grm3* and *Grin3a*) [67], [68].

In conclusion, using a variety of cell proliferation, cell cycle, cell death, cell differentiation and global transcriptome assays, we show that the daily supply of FGF-2/EGF is critical to maintain the survival and undifferentiated state of NSCs derived from the mouse olfactory bulb (particularly those cells from adult mice). Indeed, reducing the frequency of FGF-2/EGF administration from 2 to 4 days induces cell death, cell protection and cell differentiation, indicative of a loss of stem cell features and viability. Among the genes affected, *Dscaml1*, *S100b*, *Cryab*, *Pla2g7*, *Gpr17* and *Ndrg2* expression was upregulated, and that of *Calbindin* was downregulated, possibly representing the earliest molecular responses of NSCs to partial FGF-2/EGF starvation.

### Accession Number

The data discussed in this publication have been deposited in NCBI’s Gene Expression Omnibus (Edgar et al., 2002) and are accessible through GEO Series accession number GSE37516 (http://www.ncbi.nlm.nih.gov/geo/query/acc.cgi? acc = GSE37516).

## Supporting Information

Figure S1 **Effect of VEGF-C, FGF-8 and SHH on the proliferation and differentiation of aOBSCs.** Cultured aOBSCs (prepared from 4- and 6-month old mice) supplemented daily with FGF-2/EGF (20 ng/ml each) were treated with VEGF-C (V), FGF-8 or SHH for 3–5 days. BrdU (5 µM) was added for 1 h on the last day in culture to label proliferating cells (**A–D**). The images show representative fields of aOBSCs maintained with EGF/FGF-2 alone (**A**) or in combination with 20 ng/ml VEGF-C (V20; **B**), 50 ng/ml FGF-8 (**C**) or 100 ng/ml SHH (**D**), which were immunostained with BrdU antibody and stained with DAPI. (**E, F**) Cells treated with EGF/FGF-2 alone or in combination with 10 and 20 ng/ml VEGF-C were seeded under differentiation conditions, fixed after 3 days and immunostained with a TuJ1 antibody. Graphs show the percentages of proliferating BrdU^+^ cells (**G**) and differentiating TuJ1^+^ cells (**H**), and the results represent the mean ± SEM from 3–7 cultures. No significant differences were detected for any treatment group. Scale bars (F) = 75 µm (A–D) and 40 µm (E, F).
(TIF)

Figure S2 **The influence of the frequency of FGF-2/EGF addition on the OBSC cell cycle in function of the time in culture.** Embryonic OBSCs (**A–C**) and adult OBSCs (prepared from 6-month old mice; **D–F**) were grown as neurospheres, as described in Fig. 1. After 2, 3 or 4 DIV for eOBSC cultures or 5, 6 or 7 DIV for aOBSC cultures, the cells were fixed with ethanol, stained with PI and analyzed by flow cytometry. The graphs show the percentage of cells in the different phases of the cell cycle (G_0_/G_1_, **A and D**; S, **B and E**; G_2_/M, **C and F**) and the results represent the mean ± SEM from 2 cultures. The addition of growth factors at different intervals had no consistent effect on the cell cycle parameters tested over the time of eOBSC culture. However, the percentage of aOBSC cells in G_0_/G_1_ when they were partially deprived of FGF-2/EGF was greater than those in the controls at all the times analyzed.
(TIF)

Figure S3 **Decrease in the frequency of FGF-2/EGF addition progressively reduces cell viability in aOBSCs.** Embryonic (**A–D**) and adult (prepared from 6-month old mice; **E–H**) OBSCs were grown as neurospheres, as described in Fig. 1. After 1, 2, 3 or 4 DIV for eOBSC cultures or 5, 6 or 7 DIV for aOBSC cultures, the cells were collected and stained with PI and annexin V before they were analyzed by flow cytometry. Graphs show the percentage of cells in each group (viable, **A and E**; early apoptotic, **B and F**; late apoptotic, **C and G**; and dead, **D and H**) and the results represent the mean ± SEM from 2 cultures. A decrease in the frequency of growth factor addition induced a significant decrease in the number of viable cells aOBSC cultures (P<0.05 and P<0.001 for 6 and 7 DIV, respectively) over time.
(TIF)

Figure S4 **Differentiation of aOBSCs pretreated with FGF-2/EGF at different intervals during the proliferative phase.** The aOBSCs (prepared from 6-, 7- and 15-month old mice) were cultured and passaged as floating neurospheres (as described in Fig. 1) and then seeded in the absence of growth factors at a density of 100,000 cells/cm^2^ on coverslips for 3 days to induce differentiation. The cells were immunostained and stained with DAPI. Images show TuJ1^+^ (**A–C**), GFAP^+^ (**D–F**) and O4^+^ cells (**G–I**). Graphs represent the percentage of cells labeled with TuJ1 (**J**), GFAP (**K**) and O4 (**L**). Decreasing the frequency of FGF-2/EGF addition in the proliferative phase (pretreatment) produced a 20% (non-statistically significant) reduction in the percentage of neurons, yet it had no effect on the percentage of astrocytes or oligodendrocytes. Results represent the mean ± SEM from 8 cultures. Scale bars (I) = 39.73 µm.
(TIF)

Figure S5 **Differentiation of eOBSCs pretreated with FGF-2/EGF at different intervals during the proliferative phase.** The eOBSCs were cultured and passaged as floating neurospheres as described in Fig. 1. To induce differentiation, they were seeded on coverslips at a density of 100,000 cells/cm^2^ and cultured for 3 days in the absence of growth factors. The cells were then immunostained with specific antibodies and stained with DAPI. Images show representative cells labeled with TuJ1 (**A–C**), GFAP (**D–F**) and O4 (**G–I**). Graphs show the percentages of TuJ1^+^ (**J**), GFAP^+^ (**K**) and O4^+^ cells (**L**), and we found no significant differences in the percentage of cells between any treatment groups. Results represent the mean ± SEM from 8 cultures. Scale bars (I) = 39.73 µm.
(TIF)

Figure S6 **Hierarchical clustering of aOBSCs samples.** The hierarchical clustering of samples was performed using the one minus Pearson correlation metric and the average linkage method. Samples from the C4 condition clearly differ from those of the Ctr condition.
(TIF)

Figure S7 **Gene Ontology analysis of upregulated and downregulated genes in C4 versus Ctr aOBSC cultures.** (**A**) GO terms were only assessed for genes that were upregulated in C4 versus Ctr cultures in the “biological process” category. A significant enrichment was observed for the GO term “lipid catabolic process”, which includes the highly upregulated gene *Pla2g7*. (**B**) The GO analysis was also performed for downregulated genes in the C4 versus Ctr condition. As shown, genes such as *Spry4 and Adm* were annotated within one or more GO terms including “regulation of kinase activity” and “response to peptide hormone stimulus”.
(TIF)

Figure S8 **RT-qPCR analysis of mRNA levels in eOBSCs confirms upregulation of some key genes under partial FGF-2/EGF deprivation.** The graph shows the relative changes, measured by real time RT-qPCR, in mRNA levels of selected transcripts expressed in eOBSC cultures supplemented with growth factors every 2 or 4 days, as compared with cultures that were supplemented daily. Alterations were detected in *Dscaml1*, *Pla2g7*, *Gpr17*, and *Ndrg2*. Results represent the mean ± SEM of 3 experiments.
(TIF)

Figure S9 **RNA profiling analysis by microarray and RT-qPCR.** (**A**) Relative mRNA levels were determined by microarray and real time RT-qPCR for each gene from aOBSCs. Both techniques revealed similar results. (**B**) The graph shows a high correlation (coefficient of determination, R^2^ = 0.8477) between the microarray and RT-qPCR analyses of the relative mRNA levels in aOBSCs supplemented with growth factors every 4 days (C4 condition).
(TIF)

Table S1 **Primers used for the gene profile analysis of OBSCs by RT-qPCR.** The size (in base pairs, bp) of the PCR products is given and corresponded to fragments having the expected sequences.
(DOCX)
