# Supplementary figures and images for: A Global Transcriptome Analysis Reveals Molecular Hallmarks of Neural Stem Cell Death, Survival, and Differentiation in Response to Partial FGF-2 and EGF Deprivation
Source: PLoS One. 2013 Jan 7;8(1):e53594. doi: 10.1371/journal.pone.0053594 (PMC3538603; doi:10.1371/journal.pone.0053594)

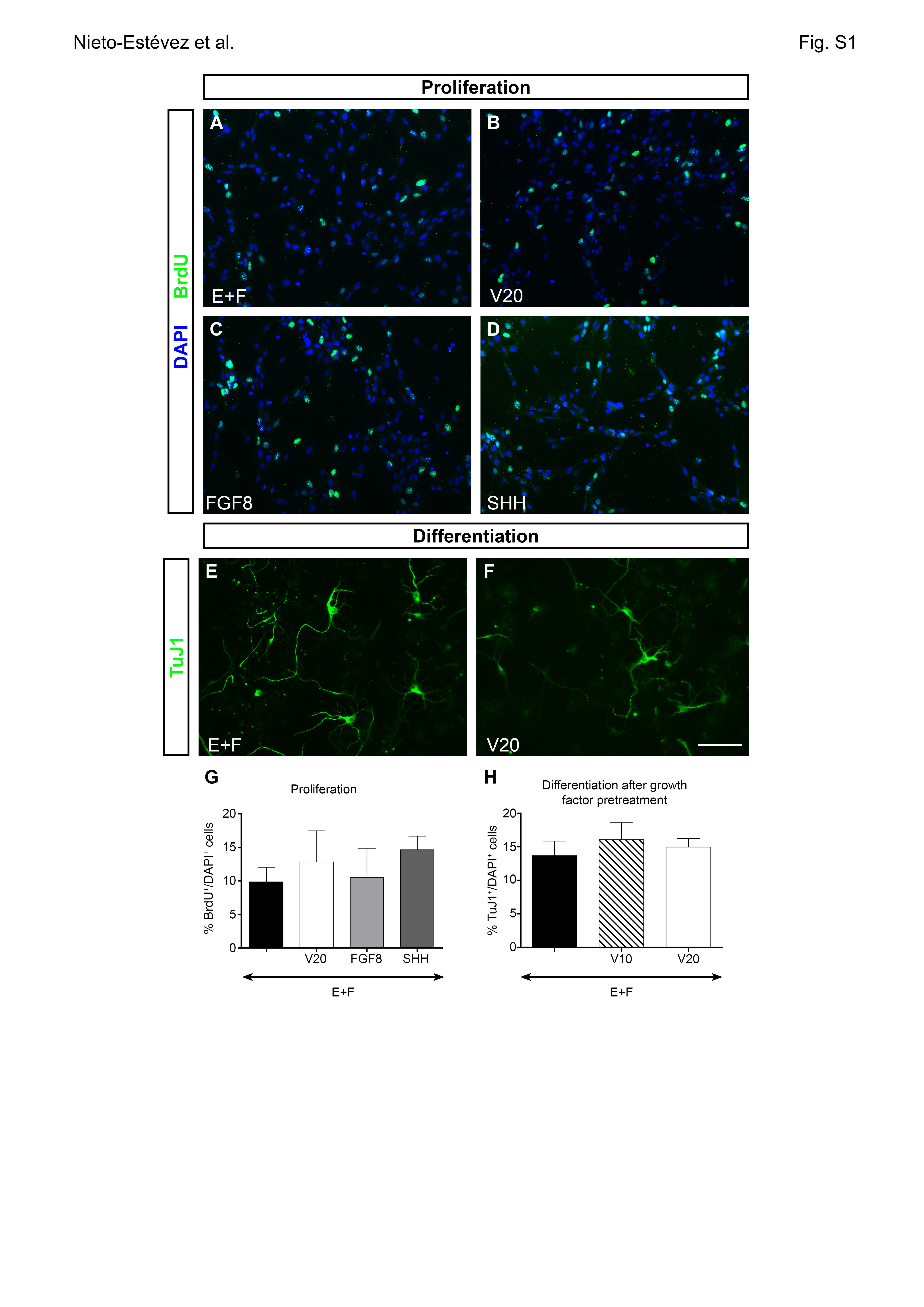

Supplement: Figure S1 — Effect of VEGF-C, FGF-8 and SHH on the proliferation and differentiation of aOBSCs. Cultured aOBSCs (prepared from 4- and 6-month old mice) supplemented daily with FGF-2/EGF (20 ng/ml each) were treated with VEGF-C (V), FGF-8 or SHH for 3–5 days. BrdU (5 µM) was added for 1 h on the last day in culture to label proliferating cells (A–D). The images show representative fields of aOBSCs maintained with EGF/FGF-2 alone (A) or in combination with 20 ng/ml VEGF-C (V20; B), 50 ng/ml FGF-8 (C) or 100 ng/ml SHH (D), which were immunostained with BrdU antibody and stained with DAPI. (E, F) Cells treated with EGF/FGF-2 alone or in combination with 10 and 20 ng/ml VEGF-C were seeded under differentiation conditions, fixed after 3 days and immunostained with a TuJ1 antibody. Graphs show the percentages of proliferating BrdU+ cells (G) and differentiating TuJ1+ cells (H), and the results represent the mean ± SEM from 3–7 cultures. No significant differences were detected for any treatment group. Scale bars (F) = 75 µm (A–D) and 40 µm (E, F). (TIF) [file pone.0053594.s001.tif]

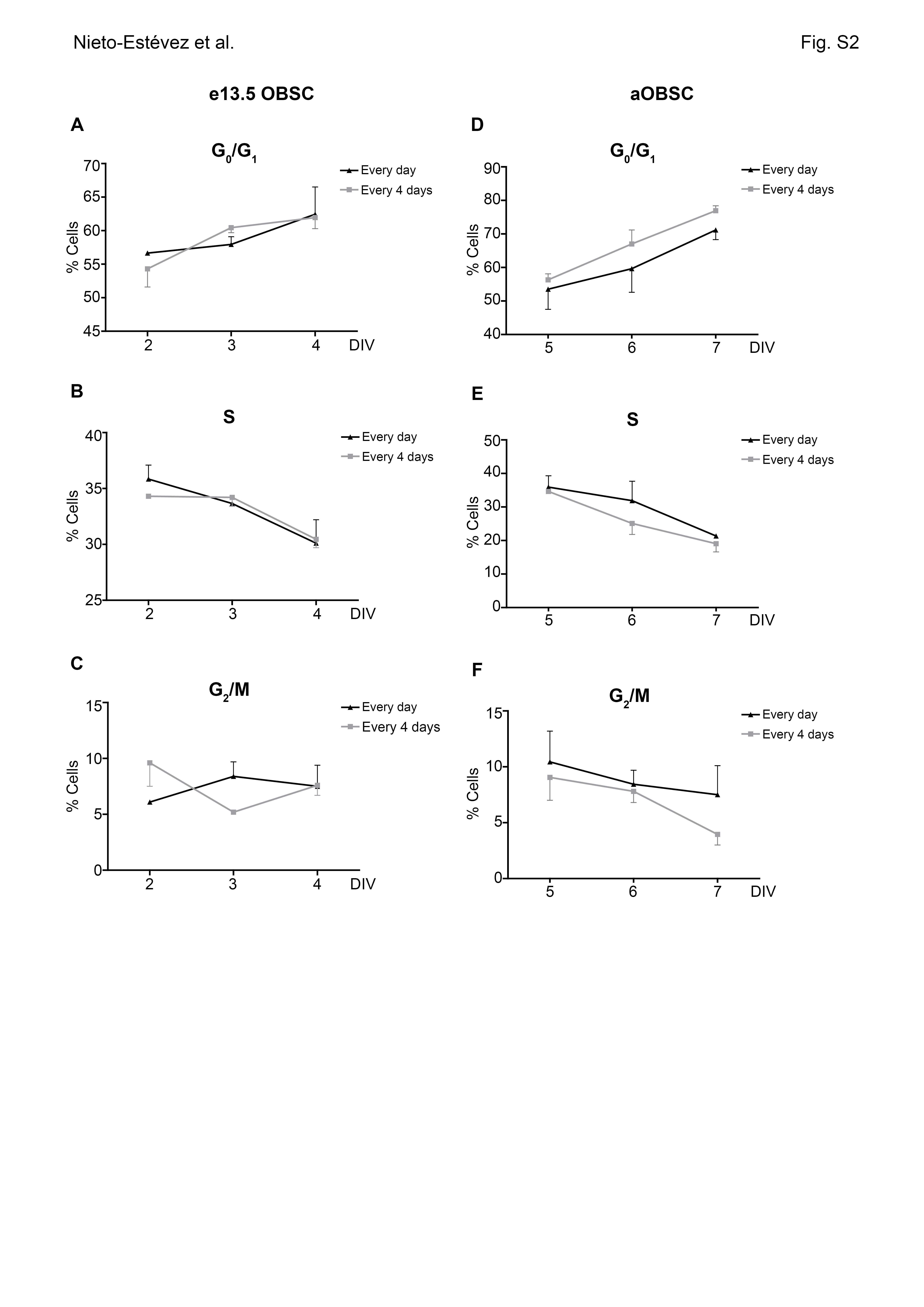

Supplement: Figure S2 — The influence of the frequency of FGF-2/EGF addition on the OBSC cell cycle in function of the time in culture. Embryonic OBSCs (A–C) and adult OBSCs (prepared from 6-month old mice; D–F) were grown as neurospheres, as described in Fig. 1. After 2, 3 or 4 DIV for eOBSC cultures or 5, 6 or 7 DIV for aOBSC cultures, the cells were fixed with ethanol, stained with PI and analyzed by flow cytometry. The graphs show the percentage of cells in the different phases of the cell cycle (G0/G1, A and D; S, B and E; G2/M, C and F) and the results represent the mean ± SEM from 2 cultures. The addition of growth factors at different intervals had no consistent effect on the cell cycle parameters tested over the time of eOBSC culture. However, the percentage of aOBSC cells in G0/G1 when they were partially deprived of FGF-2/EGF was greater than those in the controls at all the times analyzed. (TIF) [file pone.0053594.s002.tif]

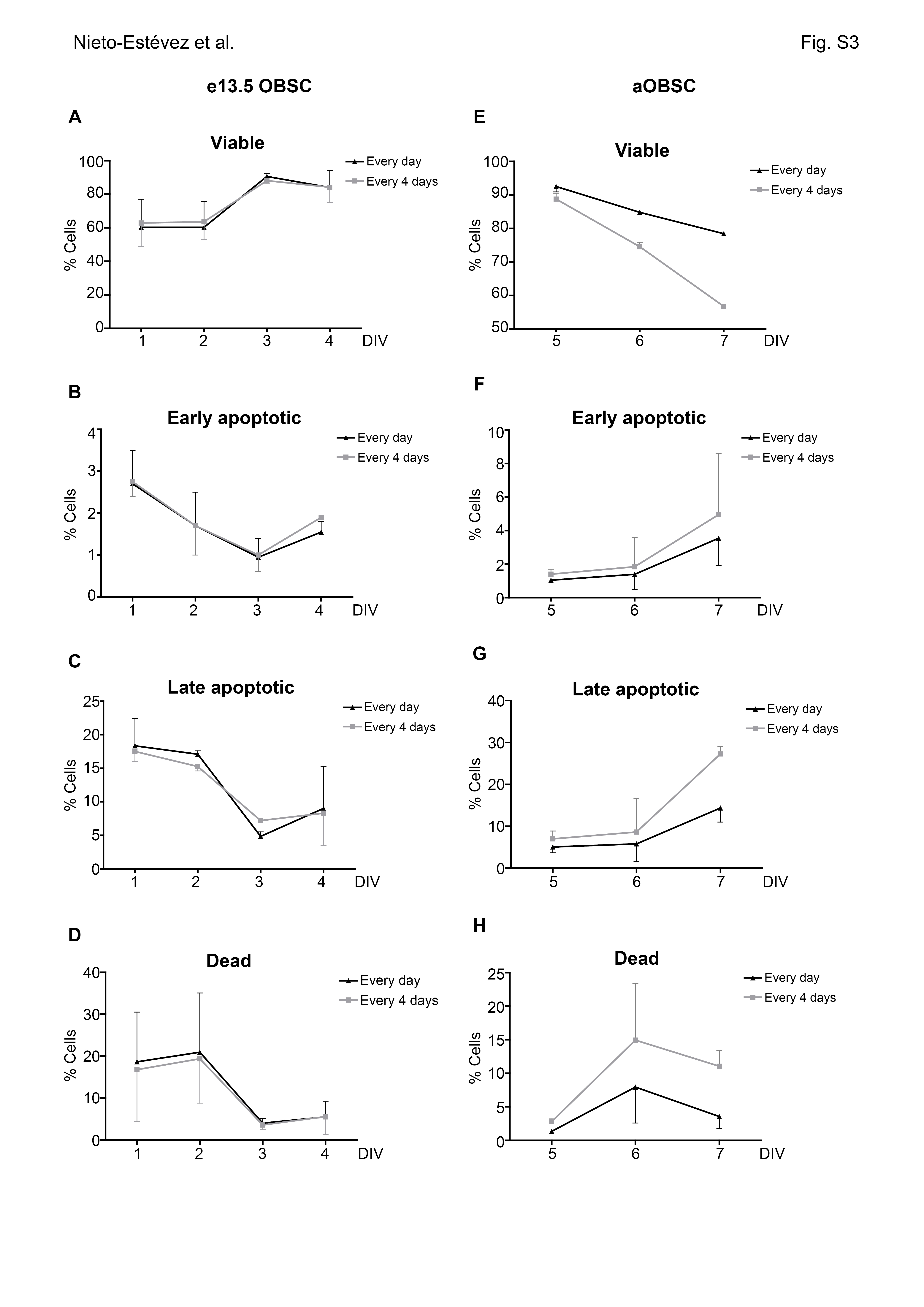

Supplement: Figure S3 — Decrease in the frequency of FGF-2/EGF addition progressively reduces cell viability in aOBSCs. Embryonic (A–D) and adult (prepared from 6-month old mice; E–H) OBSCs were grown as neurospheres, as described in Fig. 1. After 1, 2, 3 or 4 DIV for eOBSC cultures or 5, 6 or 7 DIV for aOBSC cultures, the cells were collected and stained with PI and annexin V before they were analyzed by flow cytometry. Graphs show the percentage of cells in each group (viable, A and E; early apoptotic, B and F; late apoptotic, C and G; and dead, D and H) and the results represent the mean ± SEM from 2 cultures. A decrease in the frequency of growth factor addition induced a significant decrease in the number of viable cells aOBSC cultures (P<0.05 and P<0.001 for 6 and 7 DIV, respectively) over time. (TIF) [file pone.0053594.s003.tif]

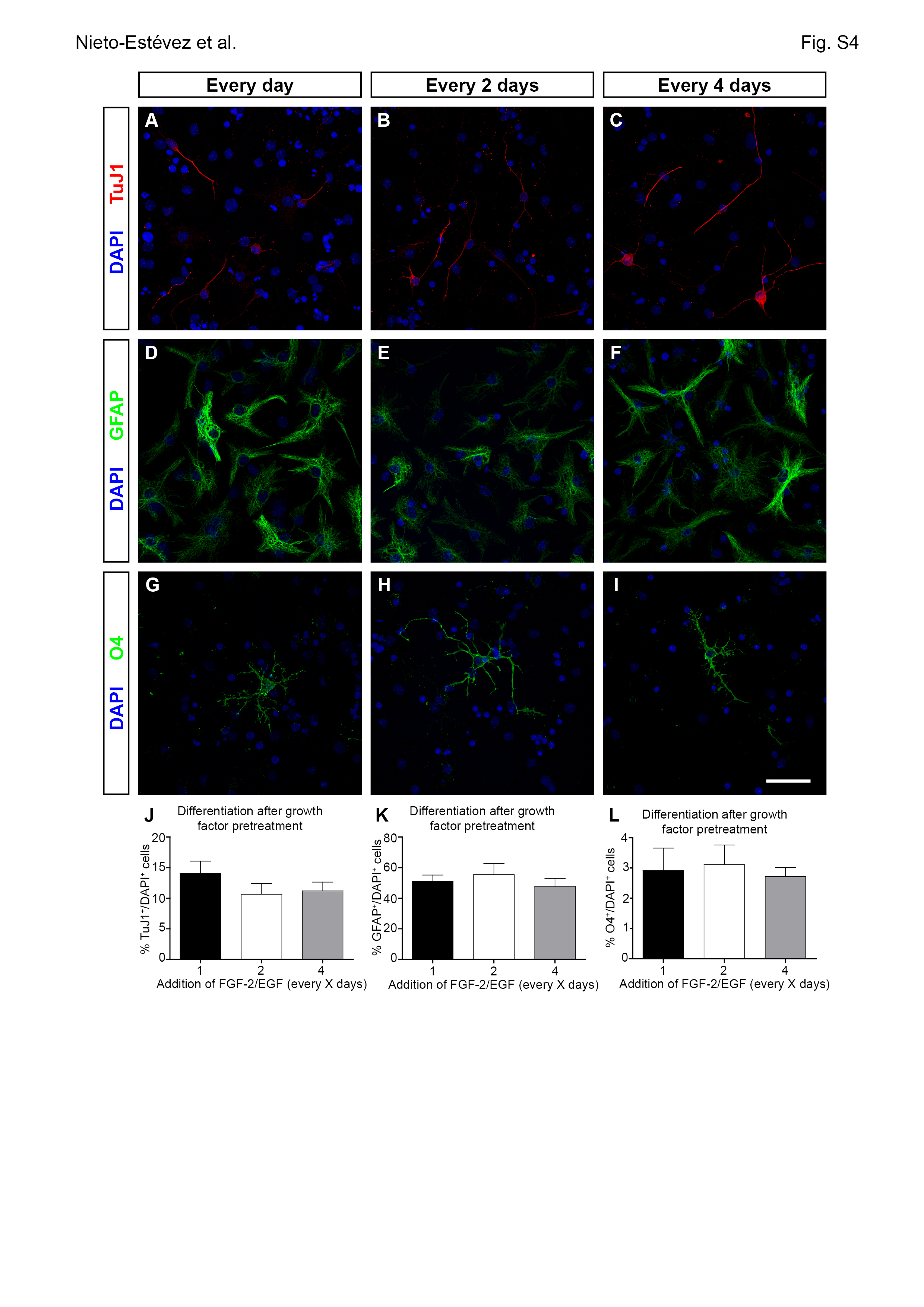

Supplement: Figure S4 — Differentiation of aOBSCs pretreated with FGF-2/EGF at different intervals during the proliferative phase. The aOBSCs (prepared from 6-, 7- and 15-month old mice) were cultured and passaged as floating neurospheres (as described in Fig. 1) and then seeded in the absence of growth factors at a density of 100,000 cells/cm2 on coverslips for 3 days to induce differentiation. The cells were immunostained and stained with DAPI. Images show TuJ1+ (A–C), GFAP+ (D–F) and O4+ cells (G–I). Graphs represent the percentage of cells labeled with TuJ1 (J), GFAP (K) and O4 (L). Decreasing the frequency of FGF-2/EGF addition in the proliferative phase (pretreatment) produced a 20% (non-statistically significant) reduction in the percentage of neurons, yet it had no effect on the percentage of astrocytes or oligodendrocytes. Results represent the mean ± SEM from 8 cultures. Scale bars (I) = 39.73 µm. (TIF) [file pone.0053594.s004.tif]

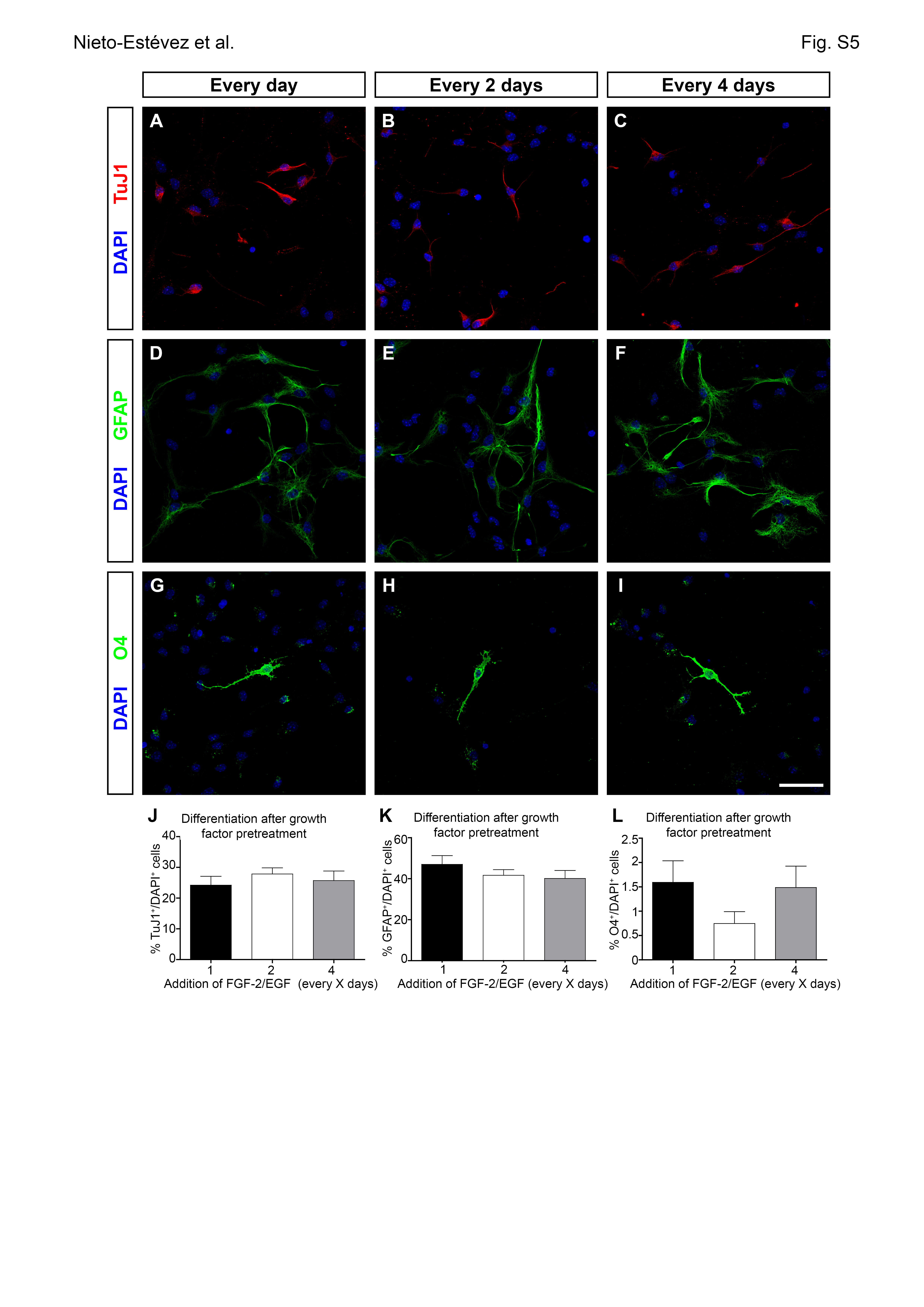

Supplement: Figure S5 — Differentiation of eOBSCs pretreated with FGF-2/EGF at different intervals during the proliferative phase. The eOBSCs were cultured and passaged as floating neurospheres as described in Fig. 1. To induce differentiation, they were seeded on coverslips at a density of 100,000 cells/cm2 and cultured for 3 days in the absence of growth factors. The cells were then immunostained with specific antibodies and stained with DAPI. Images show representative cells labeled with TuJ1 (A–C), GFAP (D–F) and O4 (G–I). Graphs show the percentages of TuJ1+ (J), GFAP+ (K) and O4+ cells (L), and we found no significant differences in the percentage of cells between any treatment groups. Results represent the mean ± SEM from 8 cultures. Scale bars (I) = 39.73 µm. (TIF) [file pone.0053594.s005.tif]

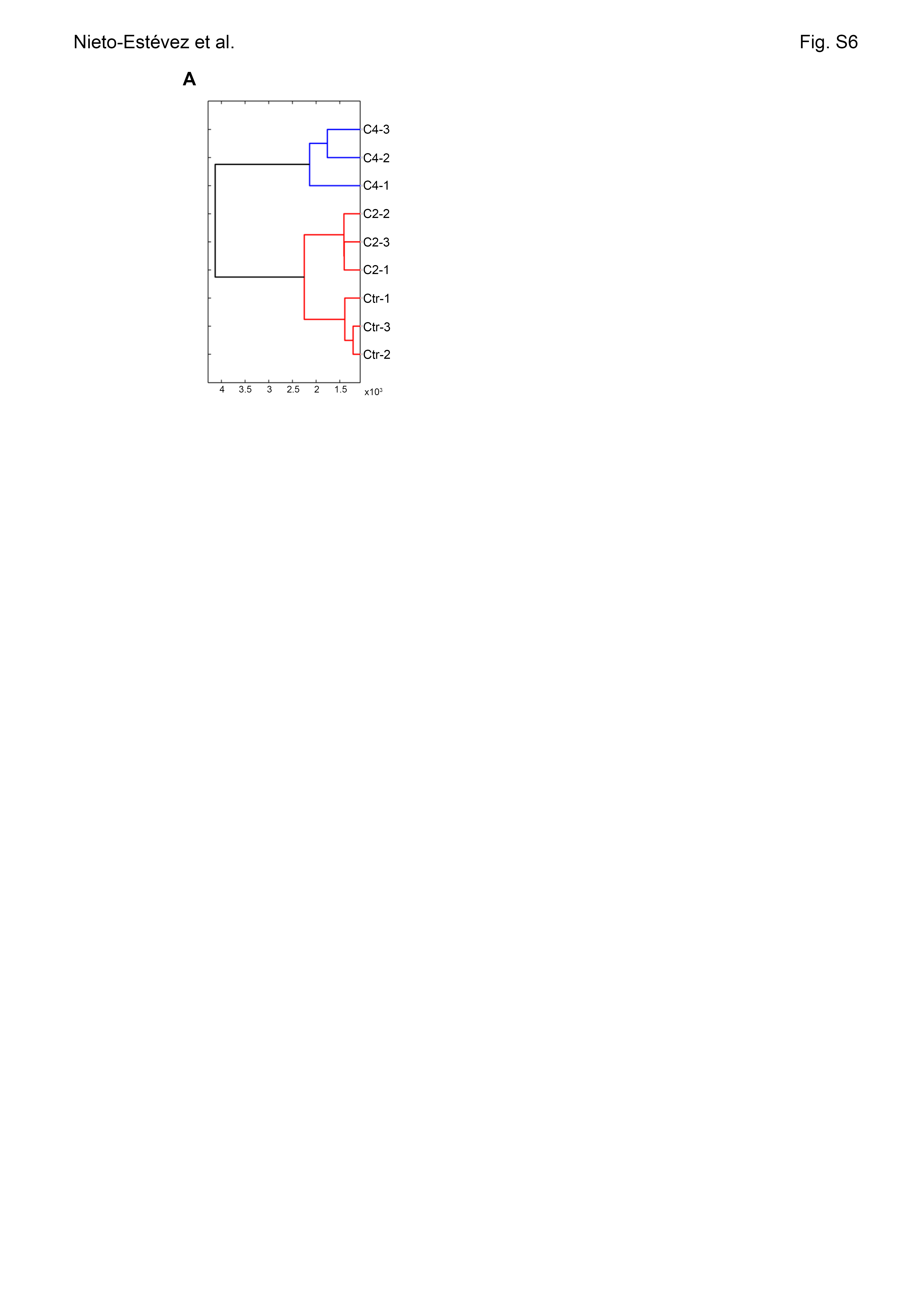

Supplement: Figure S6 — Hierarchical clustering of aOBSCs samples. The hierarchical clustering of samples was performed using the one minus Pearson correlation metric and the average linkage method. Samples from the C4 condition clearly differ from those of the Ctr condition. (TIF) [file pone.0053594.s006.tif]

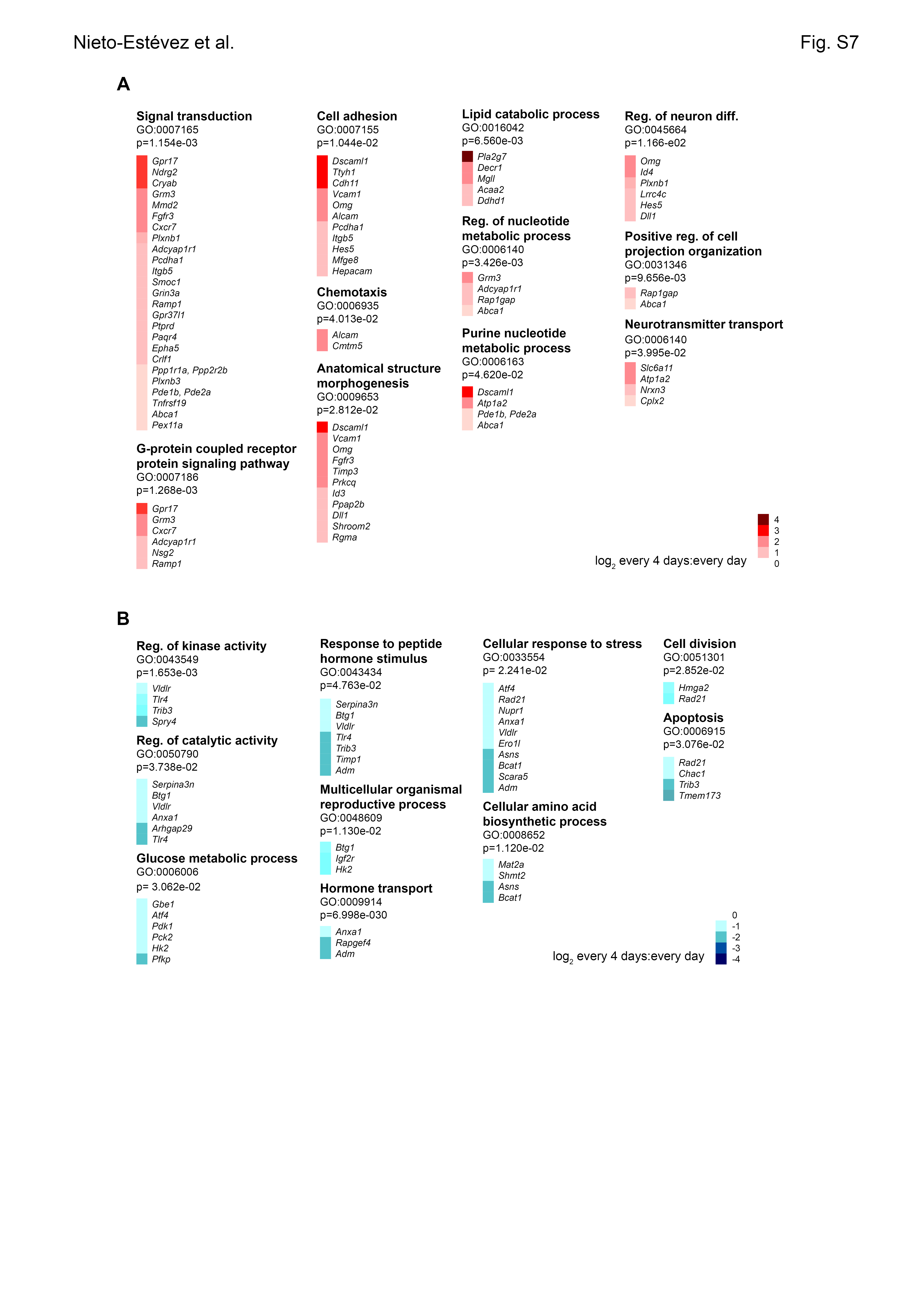

Supplement: Figure S7 — Gene Ontology analysis of upregulated and downregulated genes in C4 versus Ctr aOBSC cultures. (A) GO terms were only assessed for genes that were upregulated in C4 versus Ctr cultures in the “biological process” category. A significant enrichment was observed for the GO term “lipid catabolic process”, which includes the highly upregulated gene Pla2g7. (B) The GO analysis was also performed for downregulated genes in the C4 versus Ctr condition. As shown, genes such as Spry4 and Adm were annotated within one or more GO terms including “regulation of kinase activity” and “response to peptide hormone stimulus”. (TIF) [file pone.0053594.s007.tif]

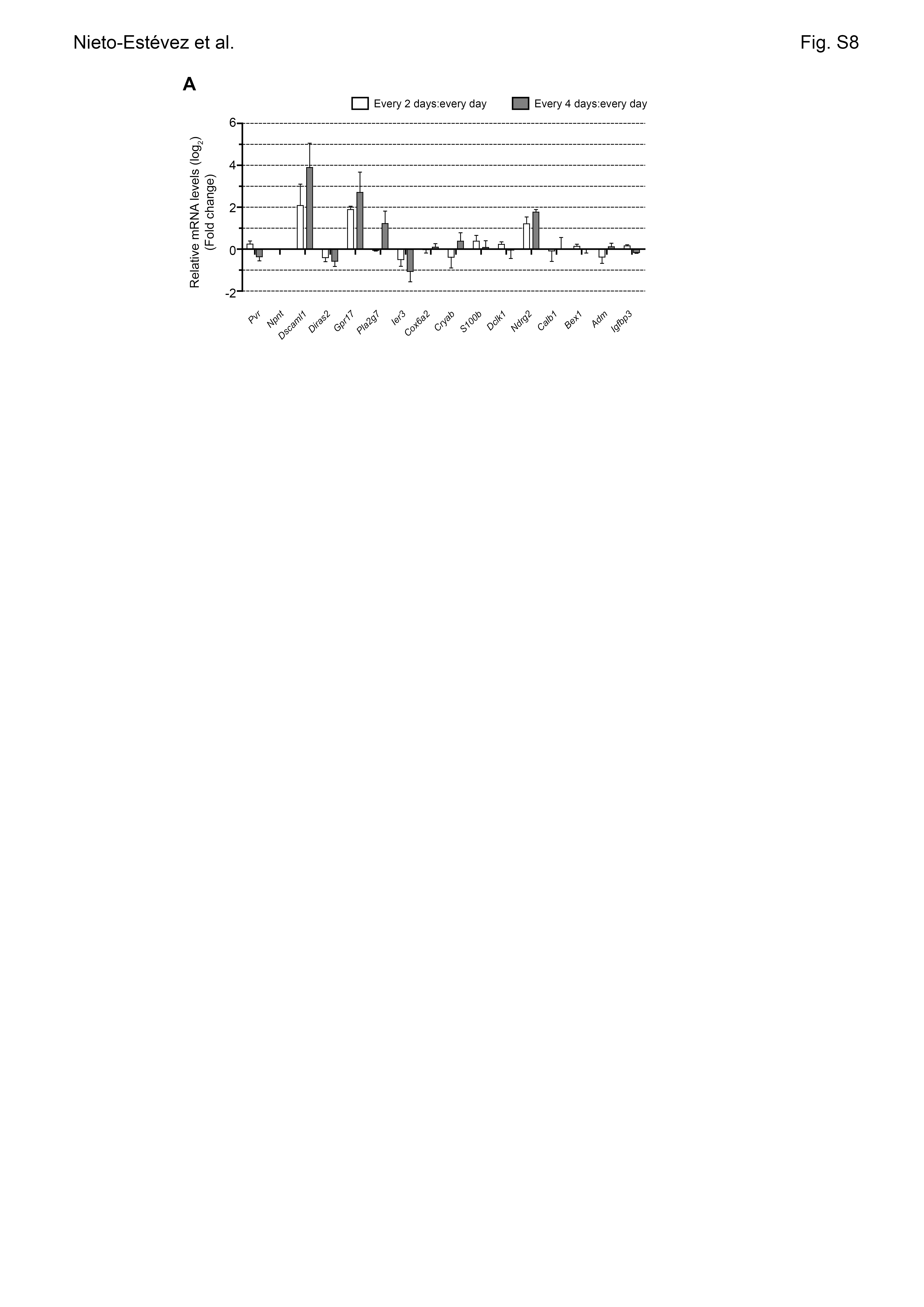

Supplement: Figure S8 — RT-qPCR analysis of mRNA levels in eOBSCs confirms upregulation of some key genes under partial FGF-2/EGF deprivation. The graph shows the relative changes, measured by real time RT-qPCR, in mRNA levels of selected transcripts expressed in eOBSC cultures supplemented with growth factors every 2 or 4 days, as compared with cultures that were supplemented daily. Alterations were detected in Dscaml1, Pla2g7, Gpr17, and Ndrg2. Results represent the mean ± SEM of 3 experiments. (TIF) [file pone.0053594.s008.tif]

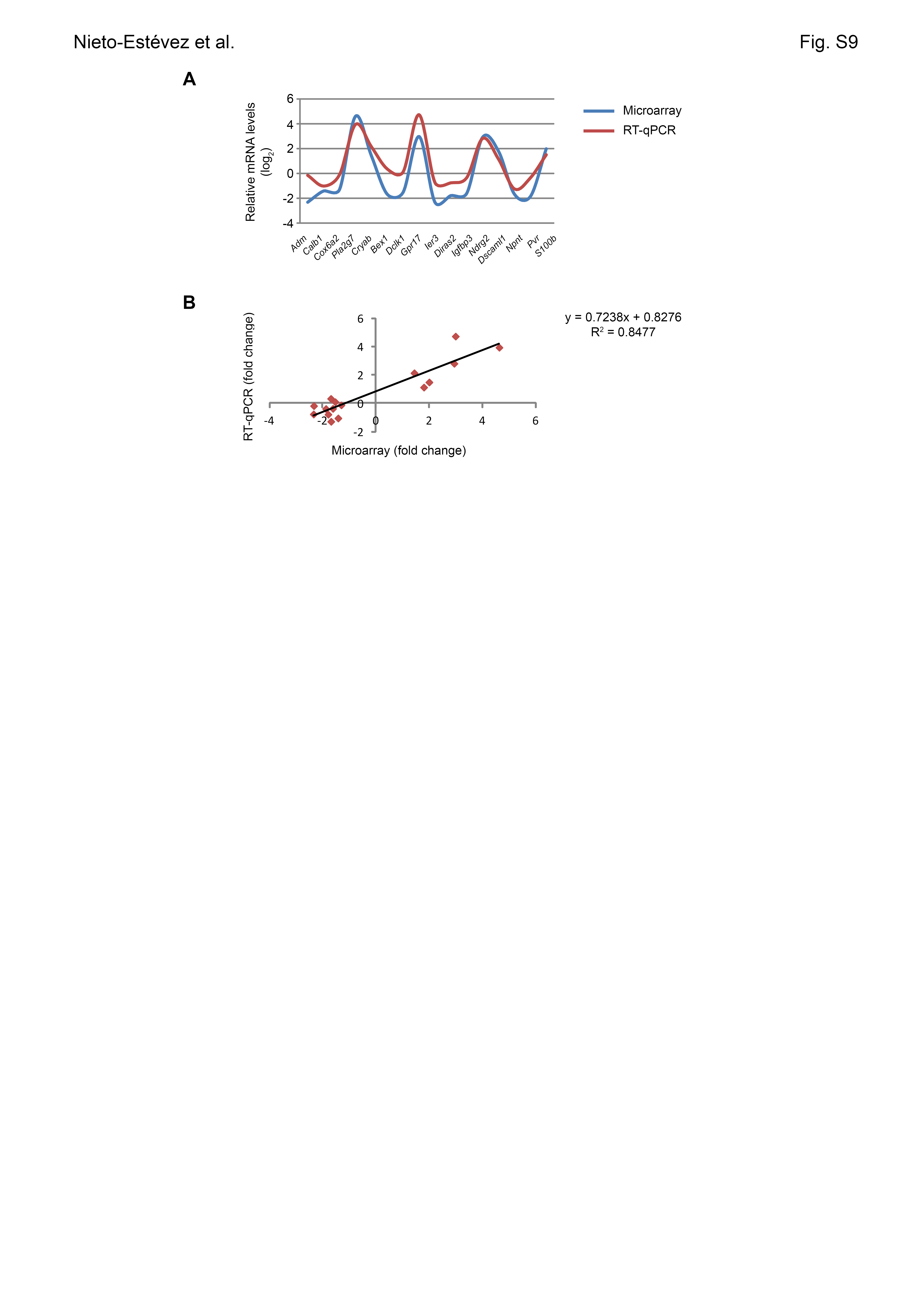

Supplement: Figure S9 — RNA profiling analysis by microarray and RT-qPCR. (A) Relative mRNA levels were determined by microarray and real time RT-qPCR for each gene from aOBSCs. Both techniques revealed similar results. (B) The graph shows a high correlation (coefficient of determination, R2 = 0.8477) between the microarray and RT-qPCR analyses of the relative mRNA levels in aOBSCs supplemented with growth factors every 4 days (C4 condition). (TIF) [file pone.0053594.s009.tif]
