# Supplementary material for: A Global Transcriptome Analysis Reveals Molecular Hallmarks of Neural Stem Cell Death, Survival, and Differentiation in Response to Partial FGF-2 and EGF Deprivation
Source: PLoS One. 2013 Jan 7;8(1):e53594. doi: 10.1371/journal.pone.0053594 (PMC3538603; doi:10.1371/journal.pone.0053594)
Supplement: Table S1 — Primers used for the gene profile analysis of OBSCs by RT-qPCR. The size (in base pairs, bp) of the PCR products is given and corresponded to fragments having the expected sequences. (DOCX) [file pone.0053594.s010.docx]

**Table S1:** Primers used for the gene profile analysis of OBSCs by RT-qPCR.

| **Gene** | **Forward primer (5’-3’)** | **Reverse primer (5’-3’)** | **Size (bp)** |
| --- | --- | --- | --- |
| *Adm* | TTGGACTTTGGGGTTTTGCTACTGT | GGAAAGCGAGTGAACCCAATAACATC | 147 |
| *Bex1* | CTCCAGCCCGCCATCCCTAAC | GCCTATCCTTGCCTTCTCCTCTTTCTA | 240 |
| *Calb1* | Qiagen Nº QT00159943 |  |  |
| *Cox6a2* | GTGCCATTCCTAGCCTCCCTTTGA | GGGAAGAGCCAGCACAAAGGTCA | 140 |
| *Cryab* | GGCACCCAGCTGGATTGACACC | CTCAATCACGTCCCCCAGAACCTT | 130 |
| *Dclk1* | CTCGGGCAAGTCACCAAGTCCATCAC | TTCCTCAGACTCTTCCCTCCTCCATC | 205 |
| *Diras2* | Qiagen Nº QT01196356 |  |  |
| *Dscaml1* | CATGTGGCTGGTAACTTTCCTCCTGC | TCACGTGCTGCAGGGAGTCATTTAC | 107 |
| *Egf* | GAGTCTGCCTGCGGATGGTA | TCTGGAGCCCTTTCGTGACAC | 273 |
| *Egfr* | GGTGCTGTAACAGAGGACAACATAGAT | GGCTGATTGTGATAGACAGGGTTC | 119 |
| *Fgf-2* | AAGCAGAAGAGAGAGGAGTTGTGTC | CCCGTTTTGGATCCGAGTTTAT | 226 |
| *Fgfr1* | CAGTGCCCTCTCAGAGACCTACG | TAACGGCTCATGAGAGAAGACAGA | 188 |
| *Fgfr2* | CGAAGACTTGGATCGAATTCTGACTC | AAGAGCTCCTTGTGTCGGGGTAAC | 107 |
| *Fgfr3* | CAAGCAGTTGGTAGAGGATTTAGAC | GTAGCAGGTCATGGGTGAACAC | 161 |
| *Gpr17* | TGTCCTTTCCTTCCTGGGTCTTCTG | GCCAGGGAGGAGTTGTCAGTCAG | 91 |
| *Ier3* | CGCGCGTTTGAACACTTCTCG | CACGGCGCTGGTAGCATCCTC | 208 |
| *Igfbp3* | CTGAGGGCGCTGCTGAATGG | ATGGAACTTGGAATCGGTCACTCG | 186 |
| *Ndrg2* | TGCCAGGACAAACACCCGAGACT | GCCATACACAGTAAAAGTGACCGAGC | 122 |
| *Npnt* | GGAGGTGGCCCAGGCAAATAGT | GGCAGCAGCATGTATCCGTTGAGA | 180 |
| *Pla2g7* | CGTGCTAGGATCTGACTCGCTCTG | CCAGAGCCGGCAGCAGACA | 264 |
| *Pvr* | ATGGCTGCTGCTGCTGTTCTGC | TCGAGATGGCCAGAGATGCGTTC | 293 |
| *S100b* | GAGGACTCCAGCAGCAAAGGTGAC | CCCGGAGTACTGGTGGAAGACATC | 98 |
| *Gapdh* | GGTGAAGGTCGGTGTGAACG | CTCGCTCCTGGAAGATGGTG | 233 |

The size (in base pairs, bp) of the PCR products is given and corresponded to fragments having the expected sequences.
